# Pharmacokinetic—Pharmacodynamic Modeling of Tumor Targeted Drug Delivery Using Nano-Engineered Mesenchymal Stem Cells

**DOI:** 10.3390/pharmaceutics13010092

**Published:** 2021-01-12

**Authors:** Shen Cheng, Susheel Kumar Nethi, Mahmoud Al-Kofahi, Swayam Prabha

**Affiliations:** 1 Department of Experimental and Clinical Pharmacology, College of Pharmacy, University of Minnesota, Minneapolis, MN 55455, USA; cheng423@umn.edu (S.C.); malkofah@umn.edu (M.A.-K.); 2 Fels Institute for Cancer Research & Molecular Biology, Lewis Katz School of Medicine, Temple University, Philadelphia, PA 19140, USA; susheel.kumar.nethi@temple.edu; 3 Department of Pharmacology, Lewis Katz School of Medicine, Temple University, Philadelphia, PA 19140, USA

**Keywords:** mesenchymal stem cells, targeted therapy, pharmacokinetics and pharmacodynamics, modeling and simulation

## Abstract

Nano-engineered mesenchymal stem cells (nano-MSCs) are promising targeted drug delivery platforms for treating solid tumors. MSCs engineered with paclitaxel (PTX) loaded poly(lactide-co-glycolide) (PLGA) nanoparticles (NPs) are efficacious in treating lung and ovarian tumors in mouse models. The quantitative description of pharmacokinetics (PK) and pharmacodynamics (PD) of nano-MSCs is crucial for optimizing their therapeutic efficacy and clinical translatability. However, successful translation of nano-MSCs is challenging due to their complex composition and physiological mechanisms regulating their pharmacokinetic-pharmacodynamic relationship (PK–PD). Therefore, in this study, a mechanism-based preclinical PK–PD model was developed to characterize the PK–PD relationship of nano-MSCs in orthotopic A549 human lung tumors in SCID Beige mice. The developed model leveraged literature information on diffusivity and permeability of PTX and PLGA NPs, PTX release from PLGA NPs, exocytosis of NPs from MSCs as well as PK and PD profiles of nano-MSCs from previous in vitro and in vivo studies. The developed PK–PD model closely captured the reported tumor growth in animals receiving no treatment, PTX solution, PTX-PLGA NPs and nano-MSCs. Model simulations suggest that increasing the dosage of nano-MSCs and/or reducing the rate of PTX-PLGA NPs exocytosis from MSCs could result in improved anti-tumor efficacy in preclinical settings.

## 1. Introduction

Since their discovery by A.J. Friedenstein in early 1970s [1], mesenchymal stem cells (MSCs) have been widely used in various therapeutic areas [2], especially in regenerative medicine [3,4]. Based on unique characteristics such as high growth and differentiation potential [5,6,7] and low immunogenicity [8,9], MSCs have been investigated in over 700 clinical trials worldwide for various clinical applications (https://clinicaltrials.gov/) [10]. MSCs exhibit specific tumor tropism in response to inflammatory signals secreted by tumor-infiltrated neutrophils and macrophages [11]. The C-X-C chemokine receptor type 4 (CXCR4)/stromal-derived factor-1 (SDF-1) axis is also involved in the recruitment of MSCs to the tumor site [12]. These mechanisms have been explored for tumor-targeted drug delivery using MSCs [13,14,15,16]. Early studies with MSCs typically involved genetic modifications to express anti–tumor proteins [17,18,19]. More recently, MSCs have been synthetically modified with polymeric nanoparticles (nano-MSCs) to enable the loading and delivery of small molecule anticancer drugs [15,16].

**Figure 1:**
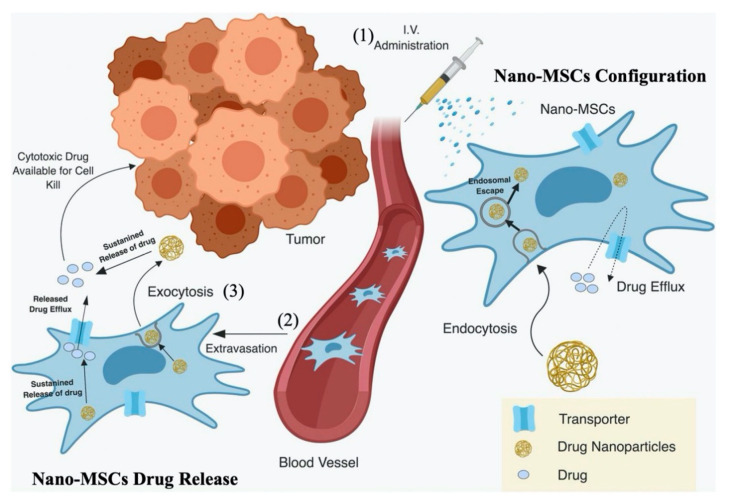
Schematic picture for the hypothetical mechanism of the action of nano-engineered mesenchymal stem cells (nano-MSCs). Following an intravenous (IV) administration of nano-MSCs (1), despite the immature release of free paclitaxel (PTX) and paclitaxel poly (lactide-co-glycolide) nanoparticles (PTX-PLGA NPs) from nano-MSCs in the plasma, PTX was rapidly carried by MSCs towards tumor-bearing sites by either active migration and extravasation or passive retention (2). PTX-PLGA NPs were then slowly exocytosed from MSCs into interstitial fluid (3). The released PTX-PLGA NPs can diffuse across the interstitial space and be taken up either by tumor cells to induce anti-tumor efficacy or by tissue cells to induce off-target effects. The PTX free drugs released from PLGA NPs inside and outside MSCs can also introduce either efficacy or toxicity. Reproduced with permission from [2], ASEPT, 2019.

Unlike other targeted therapies, nano-MSCs take advantage of the inherent tumor homing capability of MSCs, to selectively and actively deliver potent chemotherapeutic drugs to tumor sites [2]. However, overexpression of drug efflux transporters such as P-glycoprotein prevents the direct and efficient loading of soluble forms of chemotherapeutic drugs, such as paclitaxel (PTX) [2,16,20]. In order to overcome this drawback, PTX is first encapsulated into biodegradable and biocompatible poly(lactide–co–glycolide) nanoparticles (PLGA NPs). MSCs are then engineered by simple incubation with PTX-loaded PLGA NPs (PTX-PLGA NPs) to create nano-MSCs (Figure 1). Nanoengineering does not affect the morphology, size and/or phenotype of MSCs [16]. Following intravenous administration, nano-MSCs home in to tumors within 15 min of the injection and their accumulation increases over time [15,16]. PTX is released slowly from nanoparticles and there is minimal drug release during the manufacturing process (~4 h), suggesting the stability of nanoparticles inside the cells during their preparation and tumor homing [15,16]. While a fraction of loaded PTX could release inside MSCs and result in cytotoxicity, our study showed that loading PTX in MSCs results in only 8–10% cell death [16]. MSCs are resistant to PTX-induced cytotoxicity, likely because MSCs overexpress P-glycoprotein, which actively effluxes PTX out of MSCs [16]. Our group has also shown that nano-MSCs exhibit greater anti-tumor efficacy and reduced toxicity at substantially lower PTX doses, compared to that with PTX solution and PTX-PLGA NPs (9 mg/kg total dose of PTX for nano-MSCs vs. 120 mg/kg total dose of PTX for free- and nanoparticle-encapsulated drugs) [15]. However, the pharmacokinetic (PK)/pharmacodynamic (PD) relationships and the mechanism behind the superior anti-tumor efficacy of nano-MSCs have not been fully characterized.

A quantitative understanding of the dose-exposure-response relationship, via mechanism-based models, is a crucial step to optimize the preclinical efficacy of developmental therapeutics. Developed with integrated data from in vitro and in vivo studies as well as literature-derived parameter values, mechanism-based PK–PD models are able to extrapolate drug exposure and therapeutic effects, which is anticipated to reduce experimental attrition and facilitate the translation of complex therapeutics [21,22,23]. Numerous mechanistic PK–PD models have been developed over the last decade for therapies using small molecule compounds, monoclonal antibodies and antibody drug conjugates [24,25,26]. However, very few studies have focused on modeling cell-based therapies [27,28]. 

Considering there are different components responsible for the delivery of PTX to tumor cells using nano-MSCs (Figure 1), it becomes crucial to understand and characterize the biodistribution of each of these components (PTX in the form of free PTX, PLGA NPs and nano-MSCs) to achieve an optimized dosing regimen with maximum therapeutic potential. However, it is difficult to fully understand the complex processes involved in the cellular and physiological disposition of MSCs, nanoparticles and PTX, without the use of a mechanism-based PK–PD model. Therefore, the aim of this study was to develop a mechanism-based PK–PD model to characterize the time course of PTX in the form of free drug, PLGA NPs and nano-MSCs and the tumor growth profiles following nano-MSC treatment in an A549 orthotopic mouse model of lung carcinoma. The developed model will be valuable for investigating the disposition of nano-MSCs and maximizing their therapeutic potential to facilitate the translation of nano-MSCs from bench to bedside.

## 2. Materials and Methods 

### 2.1. Data 

PK (tissue drug concentrations) and PD (tumor growth over time) data were obtained from our previously published study [15], which investigated the preclinical biodistribution and efficacy of nano-MSCs in an orthotopic model of A549 lung carcinoma. The in vivo studies in mice were performed according to the protocols approval by Institutional Animal Care and Use Committee (IACUC), University of Minnesota (Protocol ID: 1605-33821A). The lung tumors were developed in female Fox Chase SCID^®^ beige mice (Charles River Laboratories) through tail vein injection of A549-luc cells and tumor growth was monitored using in vivo bioluminescence imaging as described below.

#### 2.1.1. PK Data

The tumor-bearing mice were randomized into 3 treatment groups, receiving a single 5 µg (0.25 mg/kg) intravenous (IV) injection of PTX solution, PTX-PLGA NPs, and nano-MSCs. Following the dose, mice (n = 3) were sacrificed at 2 h, and at 1-, 2-, 5- and 12-days post-treatment and blood, lung (tumor-bearing site), liver and spleen tissue samples were obtained. PTX concentrations were measured in these samples by LC/MS/MS as described before [15]. Concentrations measured as 0 in the original PK study were excluded during the model development process. 

#### 2.1.2. PD Data

Tumor growth was monitored by measuring tumor-associated bioluminescence using the IVIS Spectrum in vivo Imaging System (Caliper Life Sciences, Hopkinton, MA, USA). Treatments were initialized when tumor size reached 10^5^–10^6^ photon/sec. The tumor-bearing mice were randomized into 6 treatment groups receiving saline, untreated MSCs, MSCs with drug free blank NPs, PTX solution, PTX-PLGA NPs, or nano-MSCs. Mice in the PTX solution and PTX-PLGA NPs groups received 40 mg/kg of PTX on days 0, 4, and 8. Mice in the untreated MSCs, MSCs with blank NPs and nano-MSCs treatment groups received 1 × 10^6^ MSCs (for nano-MSCs: equivalent to 25 µg or 1.25 mg/kg PTX) on day 0 and 0.5 × 10^6^ MSCs every 14 days. For modeling purposes, animals receiving saline, untreated MSCs and MSCs loaded with blank NPs were pooled as the treatment-free group and used as control, given that PTX is the active tumor-killing cytotoxic drug. 

### 2.2. PK–PD Modeling

Nonlinear mixed-effect modeling (NONMEM^®^ version 7.4 software; ICON Development Solutions, Ellicott City, MD, USA) was performed for all analyses using the first-order (FO) and Laplacian methods for the development of the PK and PK–PD models, respectively. Since the PK study was conducted with a destructive sampling approach (one sample per subject), a naïve pooled approach was utilized. Thus, no between-subject variability (BSV) terms were estimated in the PK models, and all the variability was attributed to the residual unexplained variability (RUV). The PTX concentrations measured as ng/g were converted to ng/mL by assuming a 1 g/mL tissue density [29]. A sequential PK–PD modeling approach was employed, in which PK models for the PTX solution, PTX-PLGA NPs and nano-MSCs were developed. The estimated PK parameters were then placed into NONMEM control stream and used to drive the tumor killing in the PD model. For the BSV in PK–PD model, a log-normal distribution was assumed. For the RUV in either PK or PK–PD models, proportional error models were used and assumed to be normally distributed with mean 0 and variance σ^2^. Final NONMEM control streams for all analyses are provided in the Supporting Information. Exploratory analyses and diagnostic graphics were performed using R 3.6.3 (The R Foundation for Statistical Computing) and RStudio 1.1.453 (RStudio, Inc., Boston, MA, USA). Visual prediction checks (VPCs) and sampling importance resampling (SIR) were performed with Perl-speaks-NONMEM (PsN 4.9.0, Uppsala University, Uppsala, Sweden) under the Pirana^®^ interface [30]. 

#### 2.2.1. PK Models

**Figure 2:**
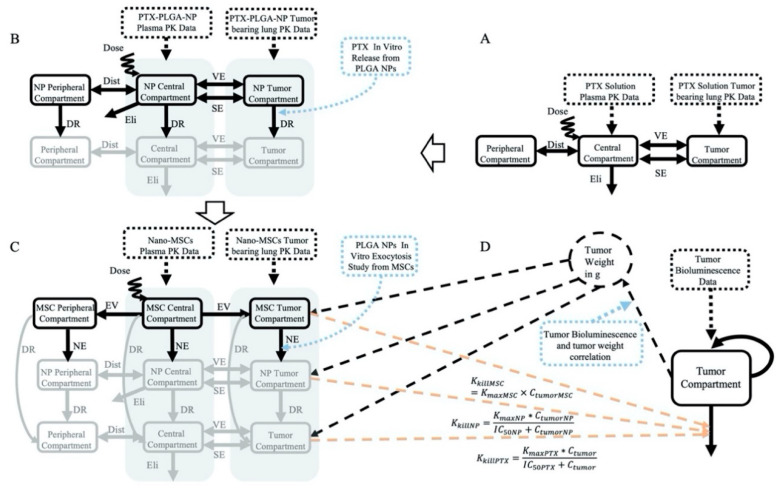
The structures of the PK models for the PTX solution (**A**), PTX-PLGA NPs (**B**), nano-MSCs (**C**) and structure of PK–PD model (**D**). The solid rectangles and solid arrows represent model compartments and inter-compartmental mass transfer, respectively. The black dotted rectangles represent the data used for each model fitting. The gray shaded areas represent the summation of compartmental concentrations. The black dotted arrows indicate the associations between model compartments and data. The blue dotted rectangles and blue dotted arrows represent the parameters derived from in vitro/in vivo studies. The dashed lines represent connections between PK and PD models without mass transfer. Specifically, the connections between the PK model derived drug concentrations and the tumor killing processes are shown in red color. Notes: Eli: elimination; Dist: distribution; VE: vascular exchange; SE: surface exchange; DR: drug release; NE: NP exocytosis; EV: extravasation.

Following the administration of nano-MSCs, the PK of PTX is complicated by the simultaneous existence of PTX in forms of free PTX, PLGA NPs and nano-MSCs (Figure 1). In order to account for PTX co-existing in three different forms, a PK model was developed using 3 parallel layers, wherein each layer corresponds to each form of PTX. A “layer by layer” approach was applied as shown in Figure 2. A free PTX–PK model (bottom layer) was first developed using PK data of PTX solution treatment group. The estimated PK parameters were then fixed and a PTX-PLGA NPs layer (middle layer) was then grafted upon. PK data of PTX-PLGA NPs was then used to develop a PK model for PTX-PLGA NPs. The estimated PK parameters for the middle layer were then fixed and used to develop the nano-MSCs (top layer) PK model. The PK parameters in top layer were then estimated using the PK data of nano-MSCs.

##### PK Model for PTX Solution (Bottom Layer)

Figure 2A describes the PK model for the PTX solution. The PTX solution PK profiles in plasma and lung were represented by a three-compartment model. The model included a central compartment, a peripheral compartment and a tumor compartment with linear elimination from the central compartment (Eli). The model was used to simultaneously characterize the disposition of PTX in the systemic circulation and tumor-bearing lungs. The distribution of PTX between the central and peripheral compartments was represented by an inter-compartmental distribution process (Dist). The model characterized the disposition of PTX from the central compartment to the tumor compartment via vascular exchange (VE) and surface exchange (SE) processes as described previously by Shah et al. [31,32]. While the surface exchange describes the diffusion of therapeutic modalities across the tumor surface, the vascular exchange describes the permeation of therapeutic modalities across the blood vessel endothelium. The rates of these two processes were influenced by the size of therapeutic modalities and the size of tumor [33,34,35]. Due to the relatively similar scale of molecular weight of small molecule compounds, the rate constants of diffusion and permeability (*D_PTX_*, *P_PTX_*) and tumor accessible fraction (*E_PTX_*) were assumed to be the same for all small molecules as shown in Table 1. The detailed PK model equations of PTX solution are provided below (Equations (1)–(3)).

(1) $$\frac{dPTX_{central}}{dt}=CLD_{PTX}\times \left(\frac{A_{PTX_{peripheral}}}{V_{PTX_{peripheral}}}-\frac{A_{PTX_{central}}}{V_{PTX_{central}}}\right)-CL_{PTX}\times \left(\frac{A_{PTX_{central}}\;}{V_{PTX_{central}}}\right)-\frac{2\times P_{PTX}\times R_{Cap}}{R_{Krogh}{}^{2}}\times VT\times \;\left(\frac{A_{PTX_{central}}}{V_{PTX_{central}}}\times E_{PTX}-\frac{A_{PTX_{tumor}}}{VT}\right)-\frac{6\times D_{PTX}}{R_{tumor}{}^{2}}\times VT\times \left(\frac{A_{PTX_{central}}}{V_{PTX_{central}}}\times E_{PTX}-\frac{A_{PTX_{tumor}}}{VT}\right)$$

$$IC_{PTX_{central}}=Dose_{PTX}\;IC:Initial\;Condition$$

(2) $$\begin{array}{cc} \frac{dPTX_{tumor}}{dt} & =\frac{2\times P_{PTX}\times R_{Cap}}{R_{Krogh}{}^{2}}\times VT\times \left(\frac{A_{PTX_{central}}}{V_{PTX_{central}}}\times E_{PTX}-\frac{A_{PTX_{tumor}}}{VT}\right)\\ & +\frac{6\times D_{PTX}}{R_{Tumor}{}^{2}}\times VT\times \left(\frac{A_{PTX_{central}}}{V_{PTX_{central}}}\times E_{PTX}-\frac{A_{PTX_{tumor}}}{VT}\right) \end{array}$$

$$IC_{PTX_{tumor}}=0$$

(3) $$\frac{dPTX_{peripheral}}{dt}=-CLD_{PTX}\times \left(\frac{A_{PTX_{peripheral}}}{V_{PTX_{peripheral}}}-\frac{A_{PTX_{central}}}{V_{PTX_{central}}}\right)$$

$$IC_{PTX_{peripheral}}=0$$

where, *PTX_central_*, *PTX_tumor_* and *PTX_peripheral_* represent central, tumor and peripheral compartment for PTX free drug, respectively. *A_PTXcentral_*, *A_PTXtumor_* and *A_PTXperipheral_* describe the PTX amounts in central, tumor and peripheral compartment, respectively. The definitions of all the other parameters are shown in Table 1. 

##### PK Model for PTX -PLGA NPs (Middle Layer)

Figure 2B describes the integrated PK model for PTX in the form of PLGA NPs. The model accounts for both PTX free drug and PTX in the form of PLGA NPs using the similar structural model that was previously used for PTX solution. The model consists of two layers, with each layer described using a three-compartment model with a central compartment, a peripheral compartment and a tumor compartment with linear elimination from the central compartment. The two layers of model are connected by a first order drug release process. The PTX release rate constant (*K_rel_*) was derived from a separate in vitro study conducted previously [16], as described in the section *K_rel_* and *K_exo_* Estimation below. Detailed PK model equations for PTX in the form of PLGA NPs are provided below (Equations (4)–(9)).

(4) $$\begin{array}{cc} \frac{dNP_{central}}{dt} & =CLD_{NP}\times \left(\frac{A_{NP_{peripheral}}}{V_{NP_{peripheral}}}-\frac{A_{NP_{central}}}{V_{NP_{central}}}\right)-CL_{NP}\times \left(\frac{A_{NP_{central}}}{V_{NP_{central}}}\right)\;\\ & -\frac{2\times P_{NP}\times R_{Cap}}{R_{Krogh}{}^{2}}\times VT\times \left(\frac{A_{NP_{central}}}{V_{NP_{central}}}\times E_{NP}-\frac{A_{NP_{tumor}}}{VT}\right)\\ & -\frac{6\times D_{NP}}{R_{Tumor}{}^{2}}\times VT\times \left(\frac{A_{NP_{central}}}{V_{NP_{central}}}\times E_{NP}-\frac{A_{NP_{tumor}}}{VT}\right)-K_{rel}\times A_{NP_{central}} \end{array}$$

$$IC_{NP_{central}}=Dose_{PTXNP}$$

(5) $$\begin{array}{cc} \frac{dNP_{tumor}}{dt} & =\frac{2\times P_{NP}\times R_{Cap}}{R_{Krogh}{}^{2}}\times VT\times \left(\frac{A_{NP_{central}}}{V_{NP_{central}}}\times E_{NP}-\frac{A_{NP_{tumor}}}{VT}\right)\\ & +\frac{6\times D_{NP}}{R_{Tumor}{}^{2}}\times VT\times \left(\frac{A_{NP_{central}}}{V_{NP_{central}}}\times E_{NP}-\frac{A_{NP_{tumor}}}{VT}\right)-K_{rel}\times A_{NP_{tumor}}\; \end{array}$$

$$IC_{NP_{tumor}}=0$$

(6) $$\frac{dNP_{peripheral}}{dt}=-CLD_{NP}\times \left(\frac{A_{NP_{peripheral}}}{V_{NP_{peripheral}}}-\frac{A_{NP_{central}}}{V_{NP_{central}}}\right)-K_{rel}\times A_{NP_{peripheral}}\;$$

$$IC_{NP_{peripheral}}=0\;$$

(7) $$\begin{array}{cc} \frac{dPTX_{central}}{dt} & =CLD_{PTX}\times \left(\frac{A_{PTX_{peripheral}}}{V_{PTX_{peripheral}}}-\frac{A_{PTX_{central}}}{V_{PTX_{central}}}\right)-CL_{PTX}\times \left(\frac{A_{PTX_{central}}}{V_{PTX_{central}}}\right)\;\;\\ & -\frac{2\times P_{PTX}\times R_{Cap}}{R_{Krogh}{}^{2}}\times VT\times \left(\frac{A_{PTX_{central}}}{V_{PTX_{central}}}\times E_{PTX}-\frac{A_{PTX_{tumor}}}{VT}\right)\\ & -\frac{6\times D_{PTX}}{R_{Tumor}{}^{2}}\times VT\times \left(\frac{A_{PTX_{central}}}{V_{PTX_{central}}}\times E_{PTX}-\frac{A_{PTX_{tumor}}}{VT}\right)+K_{rel}\times A_{NP_{central}} \end{array}$$

$$IC_{PTX_{central}}=0$$

(8) $$\begin{array}{cc} \frac{dPTX_{tumor}}{dt} & =\frac{2\times P_{PTX}\times R_{Cap}}{R_{Krogh}{}^{2}}\times VT\times \left(\frac{A_{PTX_{central}}}{V_{PTX_{central}}}\times E_{PTX}-\frac{A_{PTX_{tumor}}}{VT}\right)\\ & +\frac{6\times D_{PTX}}{R_{Tumor}{}^{2}}\times VT\times \left(\frac{A_{PTX_{central}}}{V_{PTX_{central}}}\times E_{PTX}-\frac{A_{PTX_{tumor}}}{VT}\right)+K_{rel}\times A_{NP_{tumor}}\; \end{array}$$

$$IC_{PTX_{tumor}}=0\;$$

(9) $$\frac{dPTX_{peripheral}}{dt}=-CLD_{PTX}\times \left(\frac{A_{PTX_{peripheral}}}{V_{PTX_{peripheral}}}-\frac{A_{PTX_{central}}}{V_{PTX_{central}}}\right)+K_{rel}\times A_{NP_{peripheral}}\;$$

$$IC_{PTX_{peripheral}}=0$$

where, *NP_central_*, *NP_tumor_*, *NP_peripheral_*, *PTX_central_*, *PTX_tumor_* and *PTX_peripheral_* represent the central, tumor and peripheral compartments for PTX in the form of PLGA NPs and central, tumor and peripheral compartments for PTX free drug, respectively in PTX-PLGA NPs PK model. *A_NPcentral_*, *A_NPtumor_*, *A_NPperipheral_*, *A_PTXcentral_*, *A_PTXtumor_* and *A_PTXperipheral_* describe the PTX amounts in central, tumor and peripheral compartments for PTX in the form of PLGA NPs and for PTX free drug, respectively. The definitions of all the other parameters are shown in Table 1. 

##### PK Model for Nano-MSCs (Top Layer)

Figure 2C describes the integrated PK model for PTX in the form of nano-MSCs. The model accounts for PTX free drug, PTX in the form of PLGA NPs and PTX in the form of nano-MSCs using the similar structural model previously used for PTX solution and PTX-PLGA NPs. The model consists of three layers. The structures of the PTX free drug and PTX-PLGA NPs layers are the same as the PK model for PTX-PLGA NPs. The nano-MSCs layer is described using three compartments: a central compartment, a peripheral compartment and a tumor compartment. Two unidirectional extravasation processes connect the central compartment with the peripheral and the tumor compartment in nano-MSCs layer, assuming the extravasation of nano-MSCs follows single direction. The nano-MSC and PTX-PLGA-NP layers of model are connected via a first order exocytotic process. The PLGA NPs’ exocytotic rate constant (*K_exo_*) was derived from a separate in vitro study conducted previously [36] as described in the section *K_rel_* and *K_exo_* Estimation below. The rate constant of PTX free drug release from nano-MSCs is assumed to be the same as the PTX free drug release from PLGA NPs layer and described via a first order drug release rate constant, *K_rel_*. The assumption is based on the relatively fast efflux of PTX free drug from MSCs. Detailed PK model equations for PTX in the form of nano-MSCs are provided below (Equations (10)–(18)).

(10) $$\frac{dMSC_{central}}{dt}=-K_{ct}\times A_{MSC_{central}}-K_{cp}\times A_{MSC_{central}}-K_{rel}\times A_{MSC_{central}}-K_{exo}\times A_{MSC_{central}}\;$$

$$IC_{MSC_{central}}=Dose_{nanoMSCs}$$

(11) $$\frac{dMSC_{tumor}}{dt}=K_{ct}\times A_{MSC_{central}}-K_{rel}\times A_{MSC_{tumor}}-K_{exo}\times A_{MSC_{tumor}}\;$$

$$IC_{MSC_{tumor}}=0\;$$

(12) $$\frac{dMSC_{peripheral}}{dt}=K_{cp}\times A_{MSC_{central}}\;-K_{rel}\times A_{MSC_{peripheral}}-K_{exo}\times A_{MSC_{peripheral}}\;$$

$$IC_{MSC_{peripheral}}=0$$

(13) $$\begin{array}{cc} \frac{dNP_{central}}{dt} & =CLD_{NP}\times \left(\frac{A_{NP_{peripheral}}}{V_{NP_{peripheral}}}-\frac{A_{NP_{central}}}{V_{NP_{central}}}\right)-CL_{NP}\times \left(\frac{A_{NP_{central}}}{V_{NP_{central}}}\right)\\ & -\frac{2\times P_{NP}\times R_{Cap}}{R_{Krogh}{}^{2}}\times VT\times \left(\frac{A_{NP_{central}}}{V_{NP_{central}}}\times E_{NP}-\frac{A_{NP_{tumor}}}{VT}\right)\\ & -\frac{6\times D_{NP}}{R_{Tumor}{}^{2}}\times VT\times \left(\frac{A_{NP_{central}}}{V_{NP_{central}}}\times E_{NP}-\frac{A_{NP_{tumor}}}{VT}\right)-K_{rel}\times A_{NP_{central}}+K_{exo}\times \;A_{MSC_{central}}\;\; \end{array}$$

$$IC_{NP_{central}}=0$$

(14) $$\begin{array}{cc} \frac{dNP_{tumor}}{dt} & =\frac{2\times P_{NP}\times R_{Cap}}{R_{Krogh}{}^{2}}\times VT\times \left(\frac{A_{NP_{central}}}{V_{NP_{central}}}\times E_{NP}-\frac{A_{NP_{tumor}}}{VT}\right)\\ & +\frac{6\times D_{NP}}{R_{Tumor}{}^{2}}\times VT\times \left(\frac{A_{NP_{central}\;}}{V_{NP_{central}}}\times E_{NP}-\frac{A_{NP_{tumor}}}{VT}\right)-K_{rel}\times A_{NP_{tumor}}+K_{exo}\times A_{MSC_{tumor}}\; \end{array}$$

$$IC_{NPtumor}=0$$

(15) $$\frac{dNP_{peripheral}}{dt}=-CLD_{NP}\times \left(\frac{A_{NP_{peripheral}}}{V_{NP_{peripheral}}}-\frac{A_{NP_{central}}}{V_{NP_{central}}}\right)-K_{rel}\times A_{NP_{peripheral}}+K_{exo}\times A_{MSC_{peripheral}}\;$$

$$IC_{NP_{peripheral}}=0\;$$

(16) $$\begin{array}{cc} \frac{dPTX_{central}}{dt} & =CLD_{PTX}\times \left(\frac{A_{PTX_{peripheral}}}{V_{PTX_{peripheral}}}-\frac{A_{PTX_{central}}}{V_{PTX_{central}}}\right)-CL_{PTX}\times \left(\frac{A_{PTX_{central}}}{V_{PTX_{central}}}\right)\\ & -\frac{2\times P_{PTX}\times R_{Cap}}{R_{Krogh}{}^{2}}\times VT\times \left(\frac{A_{PTX_{central}}}{V_{PTX_{central}}}\times E_{PTX}-\frac{A_{PTX_{tumor}}}{VT}\right)\\ & -\frac{6\times D_{PTX}}{R_{Tumor}{}^{2}}\times VT\times \left(\frac{A_{PTX_{central}}}{V_{PTX_{central}}}\times E_{PTX}-\frac{A_{PTX_{tumor}}}{VT}\right)+K_{rel}\times A_{NP_{central}}+K_{rel}\times A_{MSC_{central}} \end{array}$$

$$IC_{PTX_{central}}=0\;$$

(17) $$\begin{array}{cc} \frac{dPTX_{tumor}}{dt} & =\frac{2\times P_{PTX}\times R_{Cap}}{R_{Krogh}{}^{2}}\times VT\times \left(\frac{A_{PTX_{central}}}{V_{PTX_{central}}}\times E_{PTX}-\frac{A_{PTX_{tumor}}}{VT}\right)\\ & +\frac{6\times D_{PTX}}{R_{Tumor}{}^{2}}\times VT\times \left(\frac{A_{PTX_{central}}}{V_{PTX_{central}}}\times E_{PTX}-\frac{A_{PTX_{tumor}}}{VT}\right)+K_{rel}\times A_{NP_{tumor}}+K_{rel}\times A_{MSC_{tumor}}\;\; \end{array}$$

$$IC_{PTX_{tumor}}=0\;$$

(18) $$\frac{dPTX_{peripheral}}{dt}=-CLD_{PTX}\times \left(\frac{A_{PTX_{peripheral}}}{V_{PTX_{peripheral}}}-\frac{A_{PTX_{central}}}{V_{PTX_{central}}}\right)+K_{rel}\times A_{NP_{peripheral}}+K_{rel}\times A_{MSC_{peripheral}}\;\;$$

$$IC_{PTX_{peripheral}}=0$$

In the above equations, *MSC_central_*, *MSC_tumor_* and *MSC_peripheral_* represent the central, tumor and peripheral compartments, respectively, for PTX in the form of nano-MSCs. *NP_central_*, *NP_tumor_*, *NP_peripheral_*, *PTX_central_*, *PTX_tumor_* and *PTX_peripheral_* represent the central, tumor and peripheral compartments for PTX in the form of PLGA NPs and for PTX free drug, respectively. *A_MSCcentral_*, *A_MSCtumor_* and *A_MSCperipheral_*, *A_Npcentral_*, *A_Nptumor_* and *A_Npperipheral_*, *A_PTXcentral_*, *A_PTXtumor_* and *A_PTX peripheral_* describe the PTX amounts in central, tumor and peripheral compartments for PTX in the form of nano-MSCs, PLGA NPs and PTX free drug, respectively. The definitions of all the other model parameters are presented in Table 1. 

##### *K_rel_* and *K_exo_* Estimation

To estimate *K_rel_,* free PTX release from PLGA NPs was assumed to follow a first order release process and the in vivo PTX release kinetics from PLGA NPs was assumed to be comparable to in vitro PTX release kinetics. The in vitro PTX release from PLGA NPs study conducted by Sadhukha et al. [16] was used to derive *K_rel_*. A first order association model was fitted using least square method with GraphPad Prism 7. Additionally, the efflux of free PTX from nano-MSCs was assumed to happen instantaneously and therefore drug release from PLGA NPs within the cells was considered the rate-limiting step for free PTX release from nano-MSCs. Thus, the derived *K_rel_* was used for describing free drug release from both PLGA NPs and nano-MSCs. 

To estimate *K_exo_*, exocytosis of PLGA NPs from nano-MSCs was assumed to follow a first order exocytosis process. Moreover, the in vivo PLGA NP exocytosis was assumed to be comparable to in vitro exocytosis. The in vitro NPs exocytosis study conducted by Layek et al. [36] was used to derive the rate constant for NP exocytosis (*K_exo_*). A first order decay model was fitted using least square method with GraphPad Prism 7. 

#### 2.2.2. PK–PD Model

The estimated PK parameters from free PTX, PTX-PLGA NPs and nano-MSCs–PKmodels were fixed and made available in the NONMEM control stream for the tumor growth model. The predicted PTX concentrations for free drug, PLGA NPs and nano-MSCs were assumed to inhibit tumor growth as shown in Figure 2D. Tumor inhibition caused by PTX solution and PTX-PLGA NPs was described using Emax model (Equations (19) and (20)). IC50 values for PTX solution and PTX-PLGA NPs were previously reported by in vitro cytotoxicity studies [16]. Tumor inhibition caused by PTX in the form of nano-MSCs (Equation (21)) was described using a linear model. The tumor growth was represented as an exponential tumor growth function along with the tumor inhibition caused by PTX free drug, PTX-PLGA NPs and nano-MSCs. Detailed tumor growth PD model equations are provided below (Equations (19)–(22)).

(19) $$Kkill_{PTX}=\;\frac{Kmax_{PTX}\times \left(\frac{A_{PTX_{tumor}}}{VT}\right)}{IC50_{PTX}+\frac{A_{PTX_{tumor}}}{VT}}$$

(20) $$Kkill_{NP}=\;\frac{Kmax_{NP}\times \left(\frac{A_{NP_{tumor}}}{VT}\right)}{IC50_{NP}+\frac{A_{NP_{tumor}}}{VT}}\;$$

(21) $$Kkill_{MSC}=\;Kmax_{MSC}\times \left(\frac{A_{MSC_{tumor}}}{VT}\right)$$

(22) $$\frac{dTV}{dt}=Kg0\times TV-Kkill_{PTX}\times TV-Kkill_{NP}\times TV-Kkill_{MSC}\times TV\;\;$$

$$IC_{TV}=TVBL$$

In the above equations, *Kkill_PTX_*, *Kkill_NP_* and *Kkill_MSC_* are the tumor killing rates caused by PTX free drug, PTX in the form of PLGA NPs and PTX in the form of nano-MSCs, respectively. VT represents the tumor volume and TV represents the tumor compartment with the unit consistent with tumor bioluminescence. The conversion between TV and VT was derived from a separate animal study as described in the section Correlation between Tumor Weight and Tumor Bioluminescence. The definitions of all the other PD model parameters are provided in Table 2. 

The tumor bioluminescence profiles from animals receiving no treatment, PTX solution, PTX–PLGA NPs and nano-MSCs were fitted simultaneously with a population model. In order to account for the potentially early dropouts of animals having a high tumor burden, a modified M3 method-based approach proposed by Martin et al. was utilized [37]. In this approach, the highest tumor bioluminescence measurement from each animal was set as the upper “detection” limit for that animal, while measurements below the upper limit were treated as continuous data. Tumor bioluminescence measurements that were not available due to early dropouts, were assumed to be collected as regular sampling scheme and assumed to be above the upper limit for each animal. These measurements were treated as categorical data to maximize the likelihood with respect to model parameters. This method was proposed to overcome the underestimation of treatment effects introduced by the different dropout time of animals in different treatment arms.

##### Correlation between Tumor Weight and Tumor Bioluminescence

The animal studies were conducted according to the protocols approval by Institutional Animal Care and Use Committee (IACUC) at the University of Minnesota (Protocol ID: 1605-33821A). Six to eight-week-old female Fox Chase SCID Beige mice were purchased from Charles River Laboratories. The orthotopic human lung cancer model was developed in these mice by the injection of A549-luc cells (1 × 10^6^ cells) through the intravenous (tail vein) route. The tumor bioluminescence (photon/sec) and tumor weights (g) were reported for A549 tumor mice (n = 8) before and after euthanasia, respectively. Assuming the density of tumor tissue as 1 g/mL, the relation between tumor bioluminescence in photon/sec and tumor volume in mL can be investigated. In order to prevent the existence of negative tumor volume during the PD modeling process, a power function (Equation (23)) was used to investigate the association between tumor volume and tumor bioluminescence with Microsoft Excel.

(23) $$VT=a\times TV^{b\;}$$

where, *VT* is tumor volume in mL; and *TV* is tumor bioluminescence in 10^6^ photon/s.

### 2.3. Model Evaluations

PK and PK–PD models were evaluated based on the objective function values (OFVs), the stability of the model, and goodness-of-fit plots. The precisions of the final model parameters were evaluated using relative standard error (RSE) generated from covariance steps and sampling-importance-resampling (SIR)-based 95% confidence intervals (CIs) [38,39]. The final PK–PD models were evaluated using prediction-corrected visual predictive checks (pcVPC; 1000 simulations) [40]. 

### 2.4. PK and PK–PD Model Simulations

After the development of PK model for nano-MSCs, simulations were performed following the administration of 5 µg (0.25 mg/kg) PTX equivalent nano-MSCs as a single IV bolus dose into the central compartment of the nano-MSCs layer. PK profiles of the total PTX, PTX in the form of free drug, PLGA NPs and nano-MSCs were simulated in both plasma and lung. In addition, following the development of the full PK–PD model, simulations (n = 100) were carried out for animals receiving nano-MSCs, using typical values and BSV estimated in the final models. Different dosing scenarios were simulated to reflect: (i) different dosing intervals of the same total dose (10^6^ MSCs (equivalent to 25 µg or 1.25 mg/kg PTX) on day 0 and 0.5 × 10^6^ MSCs every two weeks; 10^6^ MSCs on day 0 and 0.25 × 10^6^ MSCs every week; 10^6^ MSCs on day 0 and 0.125 × 10^6^ MSCs every 3.5 days); (ii) different doses with the same dosing interval (10^6^ MSCs on day 0 and 0.25 × 10^6^ MSCs every two weeks; 10^6^ MSCs on day 0 and 0.5 × 10^6^ MSCs every two weeks; 10^6^ MSCs on day 0 and 0.75 × 10^6^ MSCs every two weeks; 10^6^ MSCs on day 0 and 2 × 10^6^ MSCs every two weeks). The effect of different *K_rel_* (0.00425, 0.0085, 0.017 h^−1^) and *K_exo_* (0.06, 0.081, 0.1 h^−1^) values on tumor growth were also simulated with the original dosing regimen of 10^6^ MSCs on day 0 and 0.5 × 10^6^ MSCs every two weeks. All the simulations were performed with mrgsolve package in R. The median and 10th and 90th percentiles of simulated tumor bioluminescence were calculated and plotted using R.

## 3. Results

### 3.1. PK and PD Data Exploration

**Figure 3:**
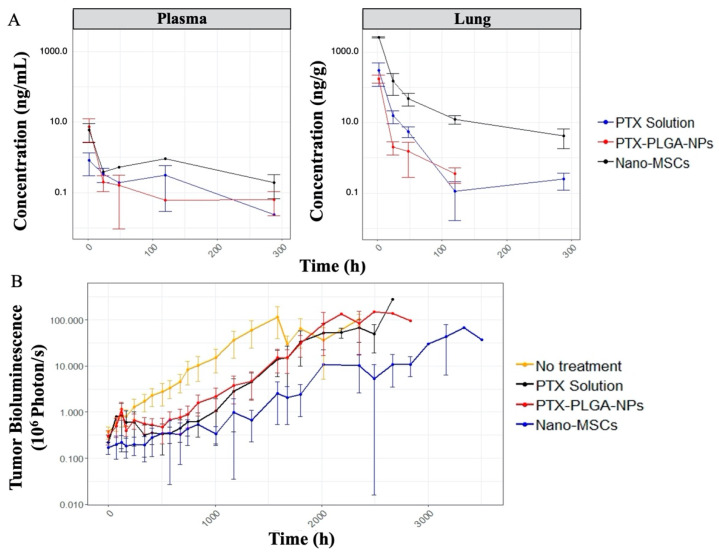
PK (A) and PD (B) data exploratory plots. The plots are on log scales. The dots and error bars represent the mean and 95% confidence intervals of either PTX concentration (**A**) or tumor bioluminescence (**B**) at each time point of each treatment group. PK profiles (**A**) is further stratified by tissues (plasma: **left**; lung: **right**).

The drug exposure in the lung (tumor-bearing tissue) following a single dose of nano-MSCs is much higher compared to that with PTX solution and PTX-PLGA NPs with comparable plasma drug exposure (Figure 3A). The tumor growth profiles are shown in Figure 3B. The relatively uneven width of 95% confidence intervals at each time point indicates relatively noisy measurements. It is noted that tumor growth profiles of animals receiving no treatments exhibit exponential growth up to around 63 days (1512 h). A plateau was observed for animals receiving no treatments after 63 days because of the death of animals with relatively high tumor bioluminescence. Similar plateaus were observed for animals in the other treatment groups. 

### 3.2. In Vitro and In Vivo Kinetics Parameter Estimation

**Figure 4:**
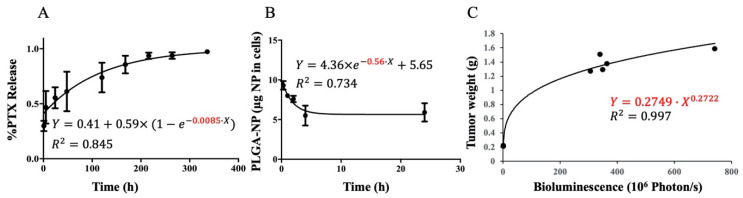
Estimations of the PTX release rate constant (**A**), PLGA NPs exocytotic rate constant (**B**) and the correlation between tumor weight and bioluminescence (**C**) with data collected from in vitro or in vivo studies. The derived PTX release rate constant (*K_rel_*) and PLGA NPs rate constant (*K_exo_*) are shown in red. The correlation estimated for tumor weight versus tumor bioluminescence is shown in red as well.

Given the limited PK data, the rate constant *K_rel_* and *K_exo_* were estimated with data acquired from in vitro drug release and NPs exocytosis studies [16,36]. The first order association model used estimated *K_rel_* as 0.0085 h^−1^ with R^2^ of 0.845 (Figure 4A), whereas the first order decay model used estimated *K_exo_* as 0.56 h^−1^ with R^2^ of 0.734 (Figure 4B). Since PK models were developed with respect to PTX mass, the estimated *K_exo_* was further multiplied by the reported PTX loading of PTX-PLGA NPs, 0.148 mg PTX/mg NP [16]. The resulted rate constant 0.083 h^−1^ was fixed in the following nano-MSCs–PK model development process. However, a fixed *K_exo_* of 0.083 h^−1^ was found to induce relatively imprecise estimates (large RSE%) of the other model parameters. Hence, we tested different *K_exo_* values ranging from 0.080 to 0.085 h^−1^ and found that fixing *K_exo_* to 0.081 h^−1^ provided a reasonable RSE% for all model parameters and gave the lowest objective function value (OFV). The association between tumor weight and tumor bioluminescence is shown in Figure 4C. 

### 3.3. PK and PK–PD Model Parameters

**Figure 5:**
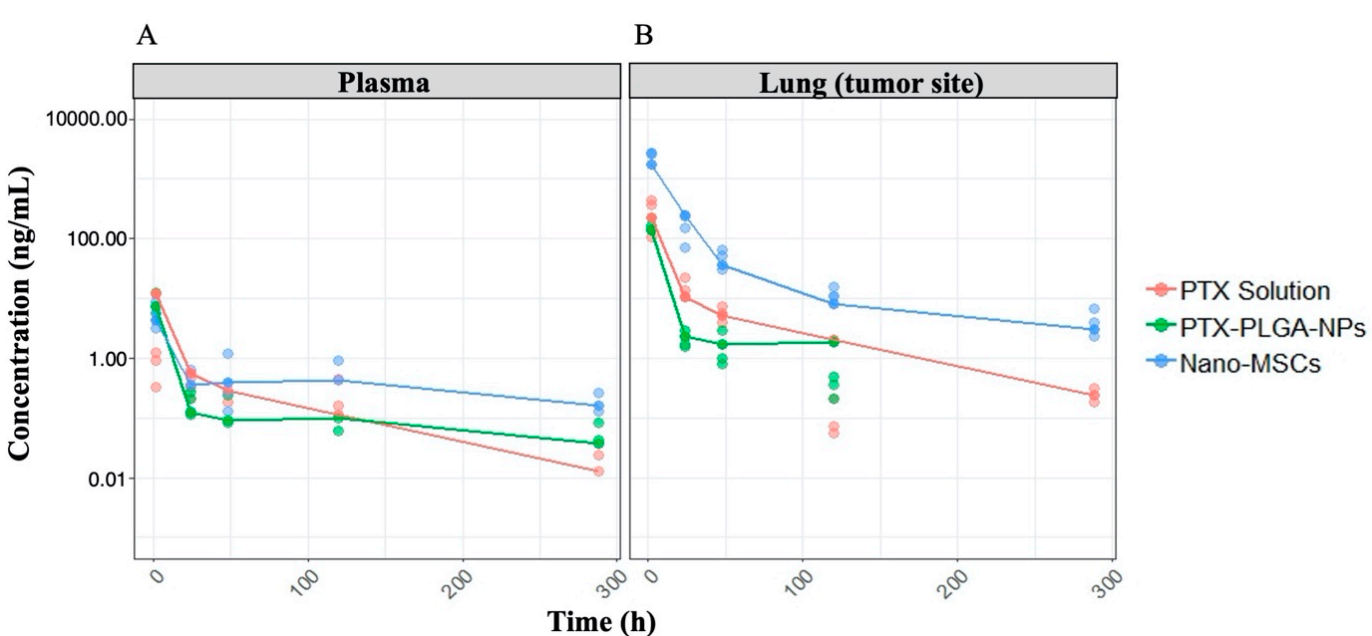
PK model fittings in plasma (**A**) and tumor-bearing lung (**B**). The plots are on log scales. The dots represent PK observations (red: PTX Solution; green: PTX-PLGA NPs; blue: nano-MSCs). The lines represent PK models predicted PK profiles (red: PTX Solution; green: PTX-PLGA NPs; blue: nano-MSCs).

The total PTX concentrations in plasma and lung following the administration of PTX solution, PTX-PLGA NPs and nano-MSCs were reasonably predicted using the developed PK models in both plasma and tumor-bearing lungs (Figure 5). The PK model parameter estimations, their RSE, SIR-based 95% CIs and sources of literature-derived parameters for PTX solution, PTX-PLGA NPs and nano-MSCs are provided in Table 1. The final PK–PD model parameter estimates, their RSE, SIR-based 95% CIs, and sources of literature-derived parameters for animals receiving no treatment, PTX solution, PTX-PLGA NPs and nano-MSCs are provided in Table 2. 

### 3.4. Model Evaluations

**Figure 6:**
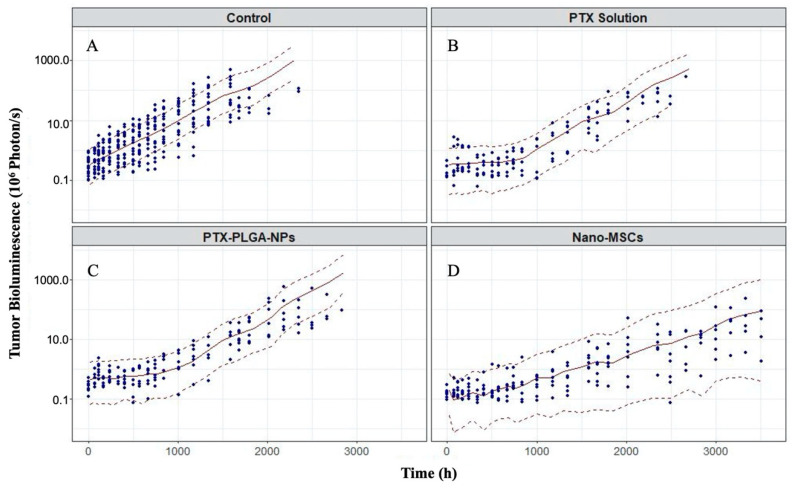
Prediction-corrected visual predictive check (pcVPC) for tumor bioluminescence–time profiles in animals receiving no treatment (**A**), PTX solution (**B**), PTX-PLGA NPs (**C**) and nano-MSCs (**D**). The plots are on log scales. The dots represent observations. The red solid lines are the median of model-predicted tumor bioluminescence profile. The red dashed lines are 10th and 90th percentiles of model-predicted tumor bioluminescence profiles, respectively.

The overall goodness-of-fit plots indicated little evidence to reject the PK–PD model (Supplementary Figures S1–S5). The prediction-corrected visual prediction check (pcVPC) was stratified by treatment groups (no treatment, treatments of PTX solution, PTX-PLGA NPs and nano-MSCs) as shown in Figure 6 on a log scale. The pcVPC suggests the developed PK–PD model is able to predict the observed tumor bioluminescence reasonably well. The final PK models and PK–PD model parameter estimates were all within their SIR-based 95% CIs (Table 2). 

**Table 1:** Parameters for pharmacokinetic model.

Parameters	Estimates (%RSE)	SIR Medians (95% CIs)	Units	Sources	Definitions
Model Associated Parameters					
*P_PTX_*	0.0875		cm/h	Literature [31,33]	Permeability rate constant for PTX free drug
*D_PTX_*	0.01		cm^2^/h	Literature [31,33]	Diffusion rate constant for PTX free drug
*E_PTX_*	0.44		unitless	Literature [31,33]	Tumor fraction accessible by PTX free drug
*R_krogh_*	0.0008		cm	Assumed [41]	Inter-capillary distance
*R_cap_*	0.0075		cm	Assumed [42]	Radius of tumor-associated capillaries
*VT*	0.3 (PK); Dynamic (PD)		mL	Assumed (PK); Dynamic (PD)	Tumor volume
*R_tumor_*	0.42 (PK); Dynamic (PD)		cm	Calculated/Assumed (PK); Dynamic (PD)	Tumor radius
*CL_PTX_*	0.909 (23%)	0.952 (0.684, 1.305)	mL/h	Estimated	Clearance for PTX free drug
*CLD_PTX_*	0.336 (68%)	0.424 (0.193, 0.712)	mL/h	Estimated	Distribution clearance for PTX free drug
*V_PTXcentral_*	6.64 (30%)	7.06 (2.81, 11.49)	mL	Estimated	Central compartment volume of distribution for PTX free drug
*V_PTXperipheral_*	18.5 (53%)	22.6 (12.6, 35.7)	mL	Estimated	Peripheral compartment volume of distribution for PTX free drug
*f_uPTX_*	0.0237 (46%)	0.0237 (0.0119, 0.0499)	unitless	Estimated	Plasma to blood ratio for PTX free drug
*P_NP_*	0.00035		cm/h	Literature [43]	Permeability rate constant for PTX in the form of PLGA NPs
*D_NP_*	3.6 × 10^−6^		cm^2^/h	Literature [44]	Diffusion rate constant for PTX in the form of PLGA NPs
*E_NP_*	0.055		unitless	Literature [33]	Tumor fraction accessible by PTX in the form of PLGA NPs
*K_rel_*	0.0085		1/h	Calculated	First order drug release rate constant for PTX free drug from PTX-PLGA NPs
*CL_NP_*	0.241 (17%)	0.259 (0.172, 0.340)	mL/h	Estimated	Clearance for PTX in the form of PLGA NPs
*CLD_NP_*	0.0627 (48%)	0.0681 (0.0349, 0.1244)	mL/h	Estimated	Distribution clearance for PTX in the form of PLGA NPs
*V_NPcentral_*	1.32 (17%)	1.41 (0.85, 1.96)	mL	Estimated	Central compartment volume of distribution for PTX in the form of PLGA NPs
*V_NPperipheral_*	43.2 (141%)	79.0 (16.6, 219.3)	mL	Estimated	Peripheral compartment volume of distribution for PTX in the form of PLGA NPs
*f_uNP_*	0.00302 (57%)	0.00325 (0.00127, 0.00684)	unitless	Estimated	Plasma to blood ratio for PTX in the form of PLGA NPs
*K_exo_*	0.081		1/h	Calculated	First order exocytosis rate constant for PTX-PLGA NPs from nano-MSCs
*K_ct_*	1.45 (22%)	1.39 (1.04, 1.84)	1/h	Estimated	Rate constant describing central to tumor compartment transfer for PTX in the form of nano-MSCs
*K_cp_*	10.2 (2%)	10.2 (9.8, 10.8)	1/h	Estimated	Rate constant describing central to peripheral compartment transfer for PTX in the form of nano-MSCs
*V_MSCcentral_*	7.15 × 10^−8^ (30%)	8.50 × 10^−8^ (2.22 × 10^−8^, 1.94 × 10^−7^)	mL	Estimated	Central compartment volume of distribution for PTX in the form of nano-MSCs
*V_MSCperipheral_*	15021 (55%)	13565 (932, 33,564)	mL	Estimated	Peripheral compartment volume of distribution for PTX in the form of nano-MSCs
Residual Unexplained Variability (RUV, proportional, CV%)					
*ε_PTX_plasma_*	116% (20%)	118.5% (67.7%, 176.6%)		Estimated	RUV for PTX solution plasma PK profiles
*ε_PTX_tumor_*	64.9% (25%)	71.5% (46.4%, 103.9%)		Estimated	RUV for PTX solution lung PK profiles
*ε_NP_plasma_*	81.2% (71%)	100.8% (50.0%, 167.6%)		Estimated	RUV for PTX-PLGA NPs plasma PK profiles
*ε_NP_tumor_*	54.5% (14%)	58.8% (42.7%, 74.0%)		Estimated	RUV for PTX-PLGA NPs lung PK profiles
*ε_MSC_plasma_*	87.3% (26%)	86.4% (73.4%, 118.5%)		Estimated	RUV for nano-MSCs plasma PK profiles
*ε_MSC_tumor_*	58.1% (22%)	61.2% (43.4%, 85.7%)		Estimated	RUV for nano-MSCs lung PK profiles

**Table 2:** Parameters for pharmacodynamic model.

Parameters	Estimates (%RSE)	SIR Medians (95% CIs)	Units	Sources	Definitions
Model Associated Parameters					
*Kg*0*_CTR_*	0.00339 (6%)	0.00339 (0.00325, 0.00352)	/h	Estimated	First order tumor growth rate constant for animals receiving no treatments
*Kg*0*_PTX_*	0.00372 (9%)	0.00367 (0.00338, 0.00397)	/h	Estimated	First order tumor growth rate constant for animals receiving PTX solution
*Kg*0*_PTXNP_*	0.00417(5%)	0.00418 (0.00394, 0.00445)	/h	Estimated	First order tumor growth rate constant for animals receiving PTX-PLGA NPs
*Kg*0*_MSC_*	0.00588 (11%)	0.00585 (0.00528, 0.00639)	/h	Estimated	First order tumor growth rate constant for animals receiving nano-MSCs
*TVBL_CTR_*	0.360 (16%)	0.365 (0.251, 0.488)	10^6^ photon/s	Estimated	Baseline tumor bioluminescence for animals receiving no treatments
*TVBL_PTX_*	0.376 (43%)	0.387 (0.208, 0.593)	10^6^ photon/s	Estimated	Baseline tumor bioluminescence for animals receiving PTX solution
*TVBL_PTXNP_*	0.539 (18%)	0.549 (0.365, 0.730)	10^6^ photon/s	Estimated	Baseline tumor bioluminescence for animals receiving PTX-PLGA NPs
*TVBL_MSC_*	0.227 (14%)	0.232 (0.173, 0.287)	10^6^ photon/s	Estimated	Baseline tumor bioluminescence for animals receiving nano-MSCs
*Kmax_PTX_*	0.00343 (26%)	0.00332 (0.00261, 0.00398)	/h	Estimated	Maximal tumor killing rate induced by PTX free drug
*Kmax_NP_*	0.000427 (372%)	0.000755 (0.000035, 0.002192)	/h	Estimated	Maximal tumor killing rate induced by PTX in the form of PLGA NPs
*K_MSC_*	4.35 × 10^−6^ (30%)	4.31 × 10^−6^ (2.79 × 10^−6^, 5.75 × 10^−6^)	/(h*(ng/mL))	Estimated	Linear tumor killing rate constant induced by PTX in the form of nano-MSCs
*IC50_PTX_*	1.5		ng/mL	Literature [16]	Concentration of PTX free drug can introduce 50% Kmax_PTX_
*IC50_NP_*	5.7		ng/mL	Literature [16]	Concentration of PTX in the form of PLGA NPs can introduce 50% Kmax_NP_
Between Subject Variability (BSV, proportional, CV%)					
*η_TVBL_*	96.4% (9%)	99.1% (77.3%, 123.6%)	Shrinkage (0%)	Estimated	BSV on baseline tumor bioluminescence
Residual Unexplained Variability (RUV, CV%)					
*ε_PTX_plasma_*	49.3% (5%)	49.5% (45.1%, 53.7%)		Estimated	RUV for tumor bioluminescence profiles in animals receiving no treatments
*ε_PTX_tumor_*	66.8% (7%)	67.4% (59.1%, 76.6%)		Estimated	RUV for tumor bioluminescence profiles in animals receiving PTX solution
*ε_NP_plasma_*	62.4% (8%)	63.0% (54.9%, 71.2%)		Estimated	RUV for tumor bioluminescence profiles in animals receiving PTX–PLGA- NPs
*ε_NP_tumor_*	62.2% (10%)	63.0% (54.8%, 72.2%)		Estimated	RUV for tumor bioluminescence profiles in animals receiving nano-MSCs

Note: * indicate multiplication.

### 3.5. Nano-MSC PK Model Simulation

**Figure 7:**
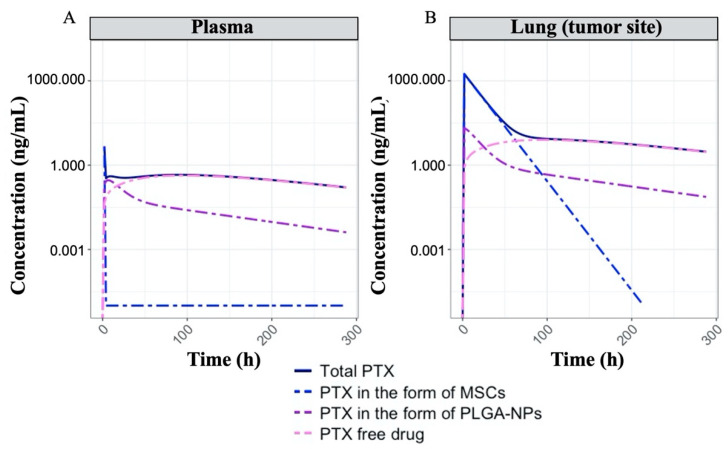
Simulated PTX concentration–time profiles in plasma (**A**) and tumor-bearing lungs (**B**) following a single IV dose of nano-MSCs administration. The plots are on log scales. On both plots, the dark-blue solid lines represent the total PTX concentrations in the respective tissues. The blue, purple and pink dash–dot lines represent the PTX concentrations in the forms of MSCs, PLGA NPs and free drug, respectively.

Simulations were performed to examine the anticipated PK profiles following the administration of nano-MSCs. Following the administration of 5 µg PTX equivalent nano-MSCs, total PTX concentration, PTX concentrations in the form of PTX solution, PLGA NPs and nano-MSCs in both plasma and lung were simulated (Figure 7). Our simulations show that following a single dose of nano-MSCs, PTX in the form of nano-MSCs quickly depleted from plasma and rapidly accumulated in the tumor-bearing lung. In the tumor-bearing lung, PTX in the form of nano-MSCs showed a mono-exponential decline. In addition, 64 h following the nano-MSC administration, free PTX starts to dominate the PTX concentration in the tumor-bearing lung. 

### 3.6. PK–PD Model Simulation

**Figure 8:**
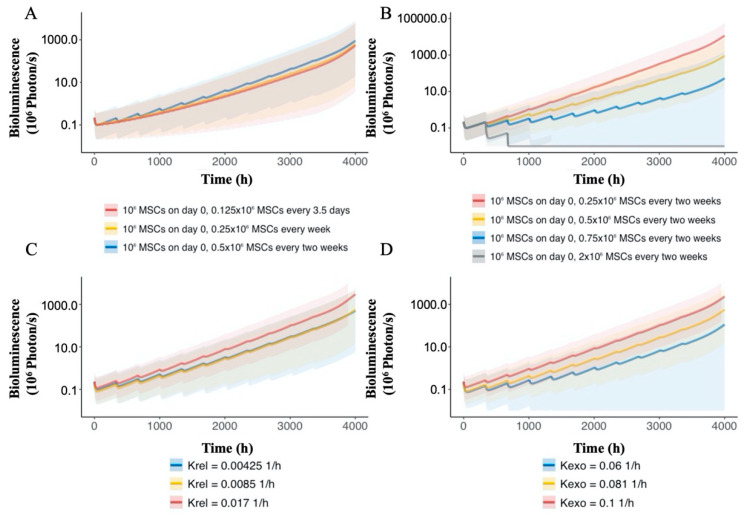
Simulated tumor bioluminescence–time profiles with different nano-MSCs dosing intervals (**A**), different nano-MSCs maintenance doses (**B**), different *K_rel_* (**C**) and different *K_exo_* (**D**). The medians of the tumor bioluminescence profiles are represented by solid lines. The areas between the 10th and 90th percentiles of the tumor bioluminescence profiles are represented by shaded areas. The colors represent the simulation conditions as shown in the respective figure legends. Note: 10^6^ nano-MSCs is equivalent to 25 μg (1.25 mg/kg) PTX.

The effect of nano-MSCs administration on tumor growth during a relevant dose regimen was examined using a simulation approach based on our PK–PD model. Our simulation suggested that different dosing intervals have little impact on the overall effectiveness of nano-MSCs (Figure 8A). On the other hand, simulation with different doses showed a significant difference in tumor growth inhibition, with the greatest inhibition driven by the highest dosage (Figure 8B). We further evaluated the impact of *K_rel_* (Figure 8C) and *K_exo_* (Figure 8D) of nano-MSCs on tumor growth. Our simulation suggested that *K_rel_* has a little impact while *K_exo_* has a significant impact on tumor growth profile. A smaller *K_exo_* is associated with a greater tumor inhibition. 

## 4. Discussion

The present study characterized the time course of free PTX, PTX-PLGA NPs and nano-MSCs and tumor growth profiles following nano-MSC dosing in an orthotopic mouse model of lung carcinoma using a nonlinear mixed-effects modeling approach. A mechanism-based PK–PD model for nano-MSCs was developed by leveraging data collected from previous in vitro and in vivo studies [15,16,36]. The developed preclinical PK models were able to adequately predict PTX exposure in animals following the administration of PTX solution, PTX-PLGA NPs and nano-MSCs in both plasma and the tumor-bearing lung. The developed preclinical PK models for PTX solution, PTX-PLGA NPs and nano-MSCs was merged with a tumor growth model to develop a PK–PD model that can characterize tumor bioluminescence–time profiles in animals receiving no treatment, PTX solution, PTX-PLGA NPs and nano-MSCs. 

Mechanistic models that account for the disposition of targeted anticancer agents in the tumor-bearing tissue are often more suitable for developing a translatable PK–PD relationship. Xie et al. [45] have reported several difficulties in building a PK–PD model with plasma drug exposure only, which points to the necessity of collecting drug concentration in tumor-bearing tissues and characterizing the relationship between drug exposure at the tumor site and tumor growth. In this study, we have developed one such model that described the disposition of PTX in the form of free drug, PTX-PLGA NPs and nano-MSCs in both plasma and tumor-bearing lungs following nano-MSC dosing. We used a destructive sampling approach to measure PTX concentrations at various tissue sites. In this approach, one animal was sacrificed per time point as previously described [15]. We used a naïve pooled modeling approach to develop PK models for PTX solution, PTX-PLGA NPs and nano-MSCs [46]. This study design allowed us to characterize the drug exposure in both plasma and tumor sites, which facilitated the development of a mechanistic PK–PD model. 

Following the administration of nano-MSCs, the PK of PTX is complicated by the simultaneous existence of PTX in forms of free PTX, PLGA NPs and nano-MSCs (Figure 1). In order to account for PTX co-existing in three different forms, a PK model was developed by merging 3 parallel PK models (layers), wherein each layer correspond with each form of PTX. The total PTX concentrations in plasma and lung following the administration of PTX solution, PTX-PLGA NPs and nano-MSCs were reasonably predicted using the developed PK models in both plasma and tumor-bearing lungs (Figure 5). In order to implement the “layer by layer” approach, several assumptions were made to describe PTX release and PLGA-NP exocytosis from nano-MSCs. PTX release from PLGA NPs and nano-MSCs were assumed to follow a first order release process. This is considered a limitation of the study since it may over-simplify the physiological conditions. A more physiologically relevant drug release kinetic model such as Higuchi model may better characterize the drug release profiles from matrix-based system such as PLGA NPs and decrease parameter imprecision [47,48]. 

Once the PK model was developed, a mechanistic PK–PD model was further developed to characterize the tumor growth in animals receiving no treatment, PTX solution, PTX-PLGA NPs and nano-MSCs, using a population modeling approach. Previous studies have frequently modeled tumor growth empirically using a mix of first order and zero order growth functions [32,49,50]. When we tried to implement similar models, convergence issues were repeatedly reported and model parameters associated with the linear growth function were estimated with insufficient precision. When we further explored tumor bioluminescence data in control (no treatment) group, we found that only few data points reflect linear growth (Figure 3B, red trace; Supplemental Figure S1, animal ID 1-18). The scarcity of data acquired in the linear (zero-order) growth phase may reflect the difference in the tumor growth pattern in different preclinical tumor models. Our model was developed using data collected from orthotopic lung tumor model rather than subcutaneous models. Orthotopic models are considered more clinically relevant than subcutaneous models [51,52]. However, the potential high aggressiveness of orthotopic tumor models [53] may cause most of animals to die before tumor volume reaches the linear growth phase. As a result, an exponential (first-order) growth function was only used to characterize tumor growth in our model.

In preclinical studies, subcutaneous tumor models are often used to study tumor growth for the purpose of optimizing the therapeutic benefits of newly developed cancer therapies. In these studies, calipers are frequently used to monitor tumor growths [31,54,55], whereas in orthotopic models, non-invasive bioluminescence measurements are needed to monitor tumor growth. Bioluminescence measurements are generally considered as a semi-quantitative method for measuring tumor size [45]. The accuracy of measurements can be compromised by numerous intrinsic and extrinsic factors such as the administration routes of substrate and cellular environments [45,56]. As a result, bioluminescence measurements are typically noisy (Figure 3B) and difficult to describe using a tumor growth PD model. This was noticeable during the development of our PK–PD model. We experienced difficulties in achieving model convergence. This is partially explained by excessive experimental noise introduced by bioluminescence measurements. The inclusion of a variability term on parameters such as exponential growth rate constant (*Kg*0) led to unsuccessful convergence of the model. As shown in Table 2, only one between subject variability (BSV) term on the baseline tumor volume was included in the final model. The failure to estimate multiple BSV terms in the final model may also reflect the difficulties in modeling tumor bioluminescence measurements. 

One limitation of the PK–PD model was that a few parameters related to PTX-PLGA NPs such as *V_NPperipheral_* and *Kmax_NP_* were estimated with relatively poor precision as shown in Table 1 and Table 2. The unexpected large relative standard error (RSE) may potentially reflect an unoptimized sampling scheme, insufficient data for model development and/or over-parameterization of the structural model. This is due to the fact that the original study was designed for the purpose of demonstrating the superior targeting and efficacy achieved by nano-MSCs and not for the purpose of PK–PD modeling. Further studies are needed to provide additional experimental PK–PD data to better characterize PTX–PLGA-NP model parameters with high precision. 

Given the complex composition of nano-MSCs, examining the PK profiles of different PTX components of this therapy experimentally is extremely difficult. Our simulations performed using the developed nano-MSCs–PK model provided mechanistic insights about the disposition of the three different forms of PTX as well as total PTX, following nano-MSCs administration. In tumor-bearing mice, following nano-MSCs administration, the relatively fast targeting, retention and extravasation of MSCs quickly deplete nano-MSCs from plasma to peripheral tissues, especially tumor-bearing sites. Nano-MSCs deliver large amount of PTX to tumor-bearing organ despite premature release of free PTX and PTX-PLGA NPs in systemic circulation (Figure 7). Subsequently, free drug and PTX-PLGA NPs released from nano-MSCs at the tumor-bearing site gradually diffuse back into the systemic circulation, which explains the shallow ascending phase following the initial rapid decline reflected in total PTX plasma concentrations after nano-MSCs dosing (Figure 3A and Figure 7A). While it would be valuable to determine the concentrations of PTX in various forms (free-PTX, PTX-PLGA NPs and PTX-MSCs), it is extremely difficult to accurately estimate the various fractions because of practical and analytical limitations. For example, separating free drug from nanoparticles requires either dialysis or ultracentrifugation. Dialysis is time consuming (nanoparticles will continue to release the drug during dialysis) while ultracentrifugation-based methods are confounded by presence of blood cells and other proteins. Because of these difficulties, most studies involving nano drug carriers report total drug concentration [57]. As more advanced analytical and separation technologies become available, future studies could analyze the concentrations of each of the forms separately to gain a better understanding of the disposition of nano-MSCs and to validate our model predictions. 

Simulations performed using the developed PK–PD model can be useful in optimizing preclinical dosing regimens for nano-MSCs. Previous studies have suggested that cancer therapies, such as gefitinib and trametinib, can achieve a better therapeutic efficacy in treating certain types of cancer when administered in multiple low doses compared with a single high dose [58,59]. However, our simulations suggest that reducing the dosing interval of nano-MSC treatment while maintaining the same total dose does not improve efficacy. In contrast, increasing the total dose of nano-MSCs while maintaining the same dosing intervals is anticipated to improve the therapeutic efficacy of nano-MSCs (Figure 8A,B). In the case of nano-MSCs, only 9 mg/kg of PTX total dose was delivered over the course of the study, which is much lower than clinically administered doses for PTX. Because our current dose is low, we do expect to be able to inject higher amounts of PTX with nano-MSCs with minimal toxicity. However, the maintenance dose of nano-MSCs is limited by the relatively large size of this therapeutic modality [60]. From our experimental experience, administrating an IV dose greater than 4 × 10^6^ nano-MSCs may increase the chance of forming cell clots within blood vessels and cause immediate death of experimental animals [15,61]. Thus, a maximal maintenance dose of 2 × 10^6^ MSCs (equivalent to 50 μg or 2.5 mg/kg PTX) was simulated. It has to be noted that if animals can tolerate such multiple doses, it would result in complete tumor remission. However, the goal should be to improve the payload capacity of nano-MSCs so as to minimize dosing frequency to further improve their effectiveness. We believe that efficacy studies conducted with the simulated maintenance doses are needed to evaluate the practicability of the predicted dosing regimens and to validate the model predictions. We expanded our simulations of the PK–PD model to examine properties that may optimize the therapeutic potential of nano-MSCs. Achieving more sustained release of chemotherapeutic drugs could improve their therapeutic efficacy [62,63]. However, in our studies, the rate of drug release from PLGA NPs had little impact on the overall tumor growth inhibition (Figure 8C). On the other hand, our model suggests that reducing the rate of exocytosis of PLGA NPs from nano-MSCs may improve nano-MSC efficacy (Figure 8D). 

## 5. Conclusions

The mechanistic model developed closely captures the complex PK–PD behavior of nano-MSCs in an orthotopic A549 lung carcinoma model. The developed model provides an opportunity to examine the effect of various parameters to further optimize the therapeutic potential of nano-MSCs. The model also provides mechanistic insights regarding the in vivo kinetics of nano-MSCs and can help guide future preclinical study design. The modeling workflow illustrated by the development of the current PK–PD model can serve as a platform for developing a mechanistic model for nano-MSCs in other tumor models. This will further help to optimize the therapeutic efficacy and clinical translatability of nano-MSCs. 

## Data Availability

The data presented in this study are available upon request.

## Supplementary Materials

The following are available online at https://www.mdpi.com/1999-4923/13/1/92/s1, Figure S1–S5: Diagnostic plots for PK–PD model. NONMEM Code for PTX solution PK model. NONMEM Code for PTX–PLGA-NP PK model. NONMEM Code for nano-MSCs–PK model. NONMEM Code for PK–PD model.
