# Supplementary material for: Pharmacokinetic—Pharmacodynamic Modeling of Tumor Targeted Drug Delivery Using Nano-Engineered Mesenchymal Stem Cells
Source: Pharmaceutics. 2021 Jan 12;13(1):92. doi: 10.3390/pharmaceutics13010092 (PMC7828117; doi:10.3390/pharmaceutics13010092)
Supplement: Supplementary file 1 [file pharmaceutics-13-00092-s001.pdf]

pharmaceutics

MDPI

Supplementary Materials: Pharmacokinetic–Pharmacodynamic Modeling of Tumor Targeted Drug Delivery Using Nano-Engineered Mesenchymal Stem Cells

Shen Cheng, Susheel Kumar Nethi, Mahmoud Al-Kofahi and Swayam Prabha*

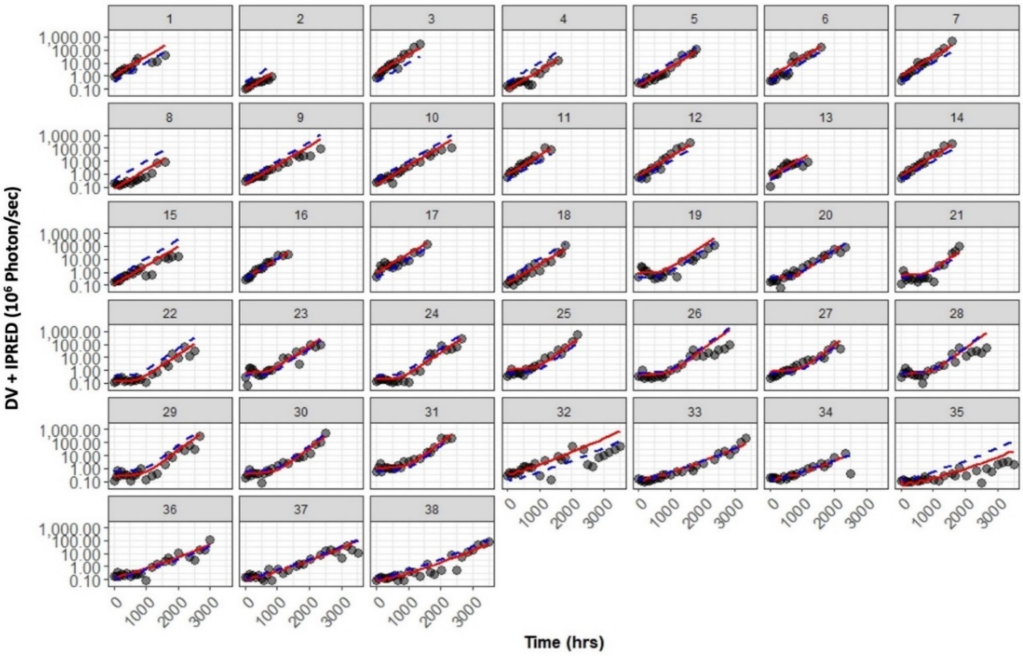

Figure S1. Individual tumor bioluminescence fittings with developed PK-PD models in animals receiving no treatments (ID: 1-18), PTX solution (ID: 19-24), PTX-PLGA-NPs (ID: 25-31) and nano-MSCs (ID: 32-38). Dots represents observed tumor bioluminescence. Red lines are the individual predictions for tumor bioluminescence profiles. Blue dashed lines are the population predictions for tumor bioluminescence profiles. Plots are on log scales.

Citation: Cheng, S.; Nethi, S.K.; Al-Kofahi, M.; Prabha, S. Pharmacokinetic–Pharmacodynamic Modeling of Tumor Targeted Drug Delivery Using Nano-Engineered Mesenchymal Stem Cells. *Pharmaceutics* **2021**, *13*, 92. https://doi.org/10.3390/pharmaceutics13010092

Received: 02 December 2020
Accepted: 11 January 2021
Published: 15 January 2021

Publisher's Note: MDPI stays neutral with regard to jurisdictional claims in published maps and institutional affiliations.

Copyright: © 2021 by the authors. Licensee MDPI, Basel, Switzerland. This article is an open access article distributed under the terms and conditions of the Creative Commons Attribution (CC BY) license (http://creativecommons.org/licenses/by/4.0/).

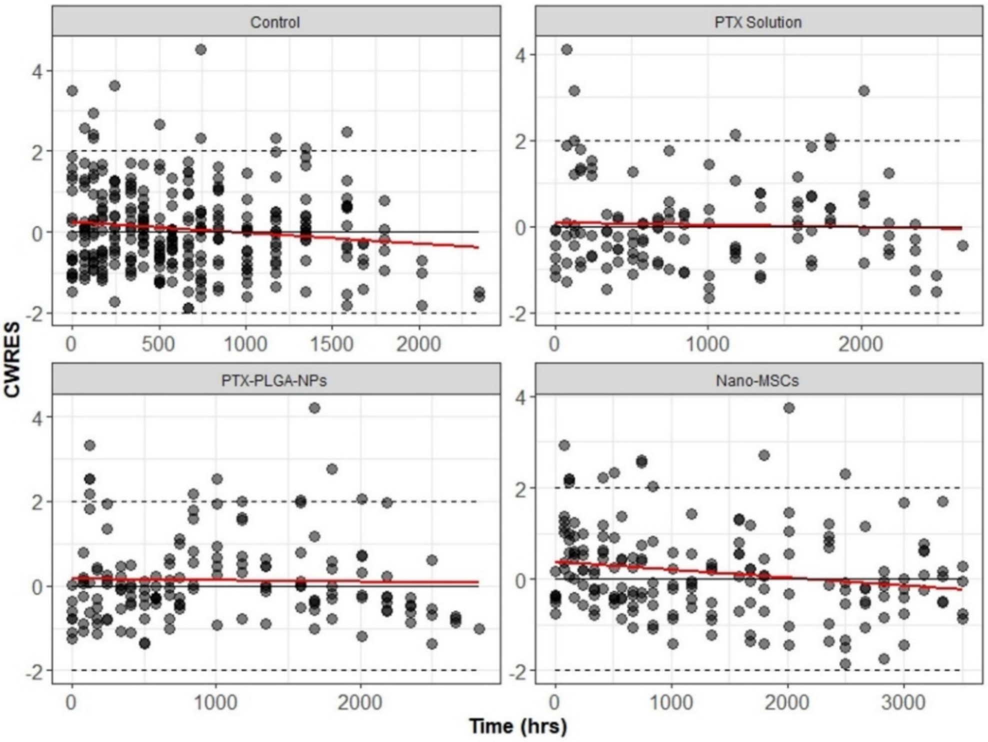

Figure S2. Conditional weighted residuals (CWRES) versus time stratified by treatment groups.

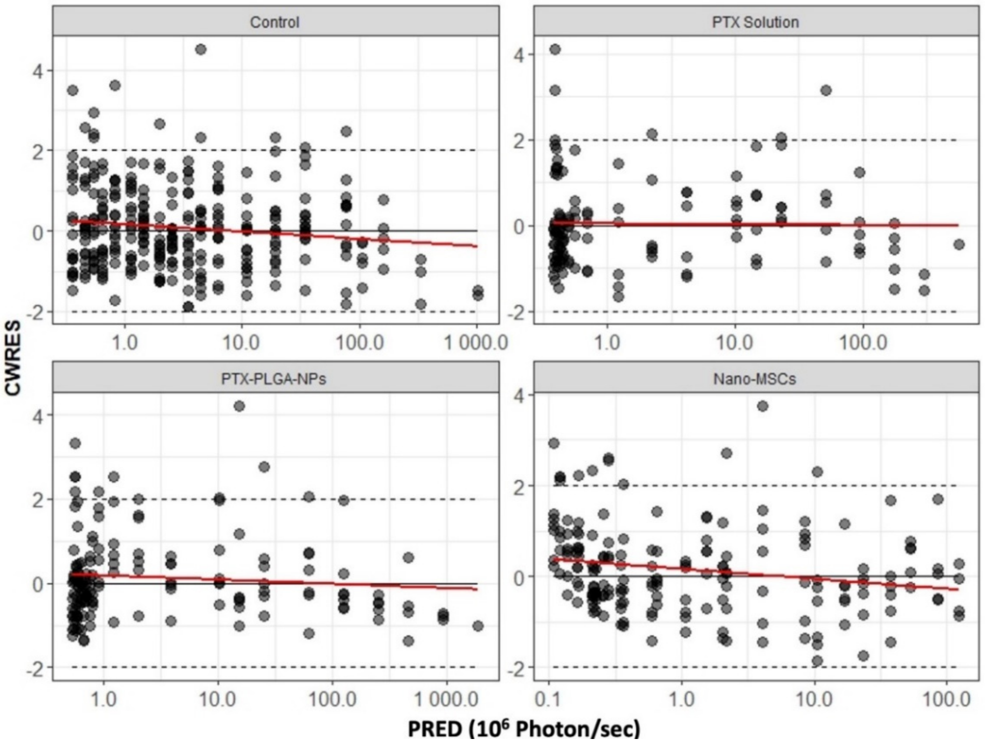

Figure S3. Conditional weighted residuals (CWRES) versus population predictions (PRED) stratified by treatment groups.

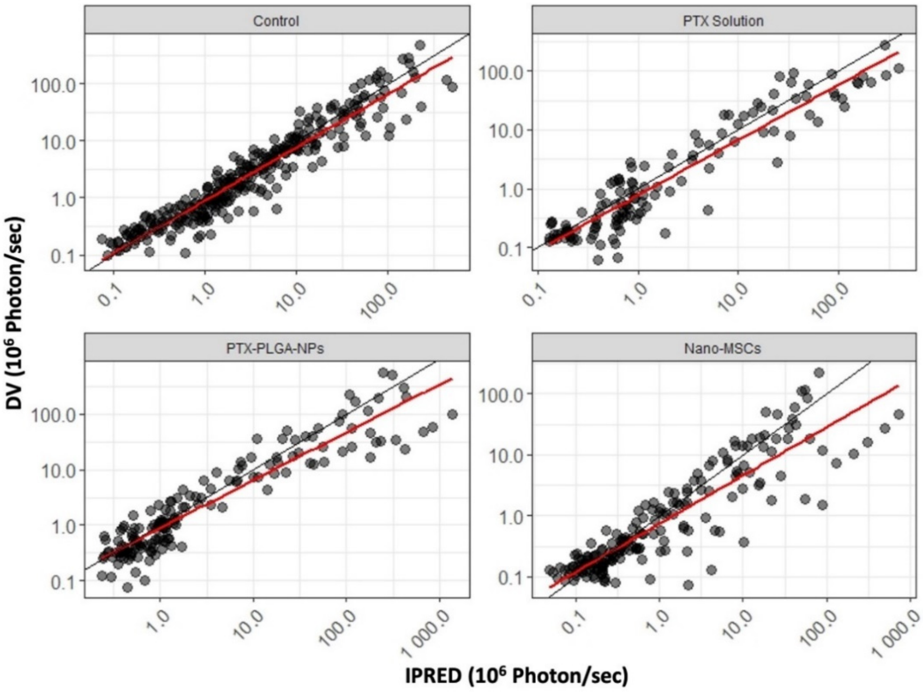

Figure S4. Dependent variables (DV) versus individual predictions (IPRED) stratified by treatment groups.

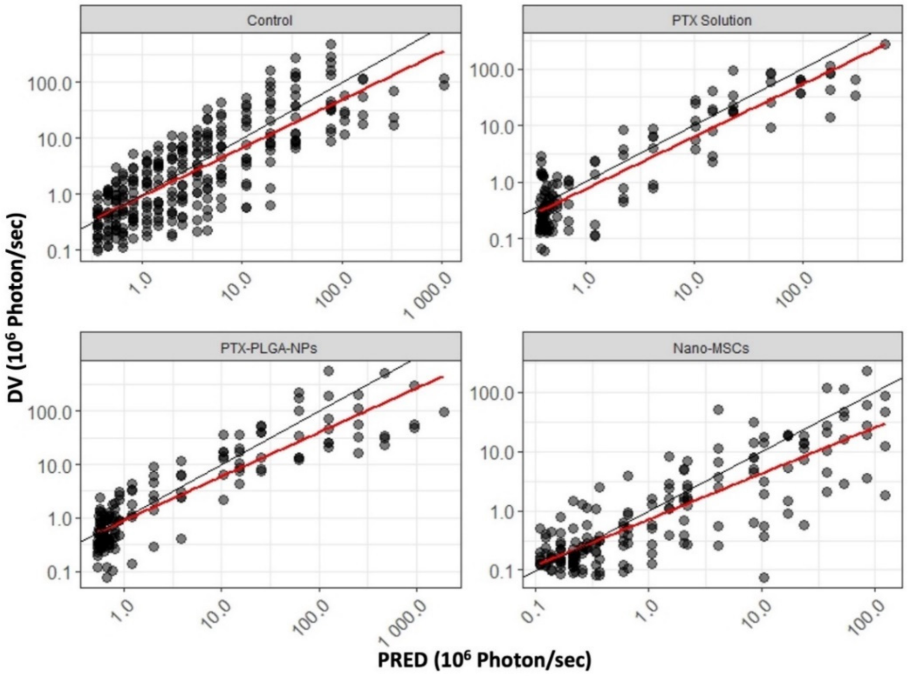

Figure S5. Dependent variables (DV) versus population predictions (PRED) stratified by treatment groups.

PTX Solution PK Model NONMEM code

Shen Cheng

11/6/2020

```
$PROB PTX Solution COMPARTMENT MODEL
$INPUT C ID TIME DV AMT CMT EVID MDV Ov
$DATA PTXNGML.csv IGNORE=C
$SUBROUTINES ADVAN13 TRANS=1 TOL=12

$MODEL NCOMP=3
COMP(CENT,DEFDOSE) ;central compartment
COMP(TUMOR)        ;tumor compartment
COMP(PHE)          ;peripheral compartment

$PK
TVCL    = THETA(1) ;typical clearance for PTX free drug
TVCLD   = THETA(2) ;typical distribution Clearance for PTX free drug
TVVPL   = THETA(3) ;typical volume of distribution of central compartment for PTX free
drug
TVVPHE  = THETA(4) ;typical volume of distribution of peripheral compartment for PTX fr
ee drug
TVFUPL  = THETA(5) ;typical plasma to blood ratio for PTX free drug
TVPDRUG = THETA(6) ;typical permeability rate constant for PTX free drug
TVDDRUG = THETA(7) ;typical diffusion rate constant for PTX free drug
TVEDRUG = THETA(8) ;typical tumor fraction accessible by PTX free drug

CL     = TVCL     ;mL/hr
CLD    = TVCLD    ;mL/hr
VPL    = TVVPL    ;mL
VPHE   = TVVPHE   ;mL
FUPL   = TVFUPL   ;unitless
PDRUG  = TVPDRUG  ;cm/hr
DDRUG  = TVDDRUG  ;cm^2/hr
EDRUG  = TVEDRUG  ;unitless

RTUMOR = 0.42     ;cm tumor radius
RKROGH = 0.0008   ;cm average distance between two tumor associated capillaries
RCAP   = 0.0075   ;cm average radius of tumor associated capillaries
VT     = 0.3      ;mL tumor volume

```

```
S1     = (1/FUPL)*VPL   ;FUPL: scaling factor PB ratio
S2     = VT

$DES
CPL      = A(1)/VPL
CT       = A(2)/VT
CPHE     = A(3)/VPHE

DADT(1) = CLD*(CPHE-CPL)-CL*CPL-(2*PDRUG*RCAP)/(RKROGH*RKROGH)*VT*(CPL*EDRUG-CT)-(6*DD
RUG)/(RTUMOR*RTUMOR)*VT*(CPL*EDRUG-CT)

DADT(2) = (2*PDRUG*RCAP)/(RKROGH*RKROGH)*VT*(CPL*EDRUG-CT)+(6*DDRUG)/(RTUMOR*RTUMOR)*V
T*(CPL*EDRUG-CT)

DADT(3) = CLD*(CPL-CPHE)

$ERROR
IF(CMT.EQ.1) THEN
IPRED = A(1)/S1
Y = IPRED*(1+ERR(1))
ENDIF

IF(CMT.EQ.2) THEN
IPRED = A(2)/S2
Y = IPRED*(1+ERR(2))
ENDIF

$THETA
(0,0.9)              ;CL   mL/hr
(0,0.2)              ;CLD  mL/hr
(0,7)                ;VPL  mL
(0,18.5)             ;VPHE mL
(0,0.0174,0.1)        ;FUPL
(0.0875 FIX)          ;PDRUG cm/hr
(0.01 FIX)            ;DDRUG cm^2/hr
(0.44 FIX)            ;EDRUG unitless

$OMEGA
1.34
0.421

$ESTIMATION METHOD=0 MAXEVAL=30000000 NOABORT NSIG=3 SIGL=12 PRINT=5 MSF=005.msf
```

```
$COV
$TABLE ID TIME DV CMT IPRED PRED RES WRES CWRES CL CLD VPL VPHE FUPL ONEHEADER NOPRINT F
ILE=sdtab005
$TABLE ID CL CLD VPL VPHE FUPL ONEHEADER NOPRINT FILE=patab005
```

PTX-PLGA-NPs PK Model NONMEM code

Shen Cheng

11/6/2020

```
$PROB PTX PLGA NPs COMPARTMENT MODEL
$INPUT C ID TIME DV AMT CMT EVID MDV OV
$DATA NPNGML.csv IGNORE=C
$SUBROUTINES ADVAN13 TRANS=1 TOL=12

$MODEL NCOMP=8
COMP(CNP)    ;central compartment for PTX in the form of PLGA-NPs
COMP(TNP)    ;tumor compartment for PTX in the form of PLGA-NPs
COMP(PNP)    ;peripheral compartment for PTX in the form of PLGA-NPs
COMP(CPTX)   ;central compartment for PTX free drug
COMP(TPTX)   ;tumor compartment for PTX free drug
COMP(PPTX)   ;peripheral compartment for PTX free drug
COMP(CTOT)   ;concentration summation for central compartments
COMP(TTOT)   ;concentration summation for tumor compartments

$PK
TVCLNP    = THETA(1)  ;typical clearance for PTX in the form of PLGA-NPs
TVCLDNP   = THETA(2)  ;typical distribution clearance for PTX in the form of PLGA-NPs
TVVPLNP   = THETA(3)  ;typical volume of distribution of central compartment for PTX in
 the form of PLGA-NPs
TVVPHENP  = THETA(4)  ;typical volume of distribution of peripheral compartment for PTX
 in the form of PLGA-NPs
TVFUPLNP  = THETA(5)  ;typical plasma to blood ratio for PTX in the form of PLGA-NPs
TVKREL    = THETA(6)  ;typical PTX release rate constant
TVPNP     = THETA(7)  ;typical permeability rate constant for PTX in the form of PLGA-NP
s
TVDNP     = THETA(8)  ;typical diffusion rate constant for PTX free drug in the form of
PLGA-NPs
TVENP     = THETA(9)  ;typical tumor fraction accessible by PTX free drug in the form of
 PLGA-NPs

CLNP   = TVCLNP    ;mL/hr
CLDNP  = TVCLDNP   ;mL/Hr
VPLNP  = TVVPLNP   ;mL
VPHENP = TVVPHENP  ;mL
FUPLNP = TVFUPLNP  ;unitless
```

```
KREL   = TVKREL    ;1/hr
PNP    = TVPNP     ;cm/hr
DNP    = TVDNP     ;cm^2/hr
ENP    = TVENP     ;unitless

RTUMOR = 0.42      ;cm tumor radius
RKROGH = 0.0008    ;cm average distance between two tumor associated capillaries
RCAP   = 0.0075    ;cm average radius of tumor associated capillaries
VT     = 0.3       ;mL tumor volume
CL     = 0.909     ;mL/hr typical clearance for PTX free drug
CLD    = 0.336     ;mL/hr typical distribution Clearance for PTX free drug
VPL    = 6.64      ;mL typical volume of distribution of central compartment for PTX fre
e drug
VPHE   = 18.5      ;mL typical volume of distribution of peripheral compartment for PTX
free drug
FUPL   = 0.0237    ;unitless typical plasma to blood ratio for PTX free drug
PDRUG  = 0.0875    ;cm/hr typical permeability rate constant for PTX free drug
DDRUG  = 0.01      ;cm^2/hr typical diffusion rate constant for PTX free drug
EDRUG  = 0.44      ;unitless typical tumor fraction accessible by PTX free drug

S1=(1/FUPLNP)*VPLNP          ;FUPLNP: scaling factor PB ratio for PTX in the form of
PLGA-NPs
S2=VT
S4=(1/FUPL)*VPL              ;FUPL: scaling factor PB ratio for PTX free drug
S5=VT

A_0(7) = 5000/S1             ;initialize concentration in concentration summation of
central compartments

$DES
CPLNP    = A(1)/VPLNP
CTNP     = A(2)/VT
CPHENP   = A(3)/VPHENP
CPL      = A(4)/VPL
CT       = A(5)/VT
CPHE     = A(6)/VPHE

DADT(1) = CLDNP*(CPHENP-CPLNP)-CLNP*CPLNP-(2*PNP*RCAP)/(RKROGH*RKROGH)*(CPLNP*ENP-CTN
P)*VT-(6*DNP)/(RTUMOR*RTUMOR)*(CPLNP*ENP-CTNP)*VT-KREL*CPLNP*VPLNP    ;PLNP

DADT(2) = (2*PNP*RCAP)/(RKROGH*RKROGH)*(CPLNP*ENP-CTNP)*VT+(6*DNP)/(RTUMOR*RTUMOR)*(CP
LNP*ENP-CTNP)*VT-KREL*CTNP*VT                                  ;TNP
```

```
DADT(3) = CLDNP*(CPLNP-CPHENP)-KREL*CPHENP*VPHENP
                                                                    ;PERINP

DADT(4) = CLD*(CPHE-CPL)-CL*CPL-(2*PDRUG*RCAP)/(RKROGH*RKROGH)*VT*(CPL*EDRUG-CT)-(6*DD
RUG)/(RTUMOR*RTUMOR)*VT*(CPL*EDRUG-CT)+KREL*CPLNP*VPLNP              ;PL

DADT(5) = (2*PDRUG*RCAP)/(RKROGH*RKROGH)*(CPL*EDRUG-CT)*VT+(6*DDRUG)/(RTUMOR*RTUMOR)*
(CPL*EDRUG-CT)*VT+KREL*CTNP*VT                                      ;T

DADT(6) = CLD*(CPL-CPHE)+KREL*CPHENP*VPHENP
                                                                    ;PERI

DADT(7) = DADT(1)/S1+DADT(4)/S4     ;SUM OF CENT COMP CONC    NO MASS TRANSFER

DADT(8) = DADT(2)/S2+DADT(5)/S5     ;SUM OF TUMOR COMP CONC    NO MASS TRANSFER

CTOTC   = A(1)/VPLNP+A(4)/VPL       ;total plasma concentration without account for BP r
atio
CTOTT   = A(2)/VT+A(5)/VT

$ERROR
CC1 = A(1)
CC2 = A(2)
CC3 = A(3)
CC4 = A(4)
CC5 = A(5)
CC6 = A(6)
CC7 = A(7)
CC8 = A(8)

IF(CMT.EQ.7) THEN
IPRED = A(1)/S1+A(4)/S4
Y = IPRED*(1+ERR(1))
ENDIF

IF(CMT.EQ.8) THEN
IPRED = A(2)/S2+A(5)/S5
Y = IPRED*(1+ERR(2))
ENDIF

$THETA
```

```
(0,0.2)                 ;CLNP    mL/hr
(0,0.1)                 ;CLDNP   mL/hr
(0,1.3)                 ;VPLNP   mL
(0,42.8)                ;VPHENP  mL
(0,0.003)               ;FUPLNP  unitless
(0.0085 FIX)            ;KREL    1/hr
(0.00035 FIX)           ;PNP     cm/hr
(0.0000036 FIX)         ;DNP     cm^2/hr
(0.055 FIX)             ;ENP     unitless

$OMEGA
0.385
0.326

$ESTIMATION METHOD=0 MAXEVAL=30000000 NOABORT NSIG=3 SIGL=9 PRINT=5 MSF=0010.msf
$COV
$TABLE ID TIME DV CMT MDV IPRED PRED CWRES CTOTC CTOTT CLNP CLDNP VPLNP FUPLNP VPHENP K
REL PNP DNP ENP VPLNP CC1 CC2 CC3 CC4 CC5 CC6 CC7 CC8 ONEHEADER NOPRINT FILE=sdtab0010
$TABLE ID CLNP CLDNP VPLNP FUPLNP VPHENP KREL PNP DNP ENP VPLNP ONEHEADER NOPRINT FILE=
patab0010
```

nano-MSCs PK Model NONMEM code

Shen Cheng

11/6/2020

```
$PROB nano-MSCs COMPARTMENT MODEL
$INPUT C ID TIME DV AMT CMT EVID MDV OV
$DATA MSCNGML.csv IGNORE=C
$SUBROUTINES ADVAN8 TRANS=1 TOL=12

$MODEL NCOMP=11
COMP(CMSC)   ;central compartment for PTX in the form of nano-MSCs
COMP(TMSC)   ;tumor compartment for PTX in the form of nano-MSCs
COMP(PMSC)   ;peripheral compartment for PTX in the form of nano-MSCs
COMP(CNP)    ;central compartment for PTX in the form of PLGA-NPs
COMP(TNP)    ;tumor compartment for PTX in the form of PLGA-NPs
COMP(PNP)    ;peripheral compartment for PTX in the form of PLGA-NPs
COMP(CPTX)   ;central compartment for PTX free drug
COMP(TPTX)   ;tumor compartment for PTX free drug
COMP(PPTX)   ;peripheral compartment for PTX drug
COMP(CTOT)   ;concentration summation for central compartments
COMP(TTOT)   ;concentration summation for tumor compartments

$PK
TVK12      = THETA(1) ;typical rate constant describing central compartment to tumor co
mpartment transfer for PTX in the form of nano-MSCs
TVK13      = THETA(2) ;typical rate constant describing central compartment to peripher
al compartment transfer for PTX in the form of nano-MSCs
TVKEXO     = THETA(3) ;typical first order exocytosis rate constant for PTX-PLGA-NPs fr
om nano-MSCs
TVVPLMSC   = THETA(4) ;typical central compartment volume of distribution for PTX in th
e form of nano-MSCs
TVVPHEMSC  = THETA(5) ;typical peripheral compartment volume of distribution for PTX in
 the form of nano-MSCs

K12    = TVK12       ;1/hr
K13    = TVK13       ;1/hr
KEXO   = TVKEXO      ;1/hr
VPLMSC = TVVPLMSC     ;mL
VPHEMSC= TVVPHEMSC    ;mL
```

```
RTUMOR = 0.42          ;cm tumor radius
RKROGH = 0.0008        ;cm average distance between two tumor associated capillaries
RCAP   = 0.0075        ;cm average radius of tumor associated capillaries
VT     = 0.3           ;mL tumor volume
CL     = 0.909         ;mL/hr typical clearance for PTX free drug
CLD    = 0.336         ;mL/hr typical distribution Clearance for PTX free drug
VPL    = 6.64          ;mL typical volume of distribution of central compartment for PTX f
ree drug
VPHE   = 18.5          ;mL typical volume of distribution of peripheral compartment for PT
X free drug
FUPL   = 0.0237        ;unitless typical plasma to blood ratio for PTX free drug
PDRUG  = 0.0875        ;cm/hr typical permeability rate constant for PTX free drug
DDRUG  = 0.01          ;cm^2/hr typical diffusion rate constant for PTX free drug
EDRUG  = 0.44          ;unitless typical tumor fraction accessible by PTX free drug
CLNP   = 0.241         ;mL/hr typical clearance for PTX in the form of PLGA-NPs
CLDNP  = 0.0627        ;mL/hr typical distribution clearance for PTX in the form of PLGA-N
Ps
VPLNP  = 1.32          ;mL typical volume of distribution of central compartment for PTX i
n the form of PLGA-NPs
VPHENP = 43.2          ;mL typical volume of distribution of peripheral compartment for PT
X in the form of PLGA-NPs
FUPLNP = 0.003         ;unitless typical plasma to blood ratio for PTX in the form of PLGA
-NPs
KREL   = 0.0085        ;1/hr typical PTX release rate constant
PNP    = 0.00035       ;cm/hr typical permeability rate constant for PTX in the form of PL
GA-NPs
DNP    = 0.0000036     ;cm^2/hr typical diffusion rate constant for PTX free drug in the f
orm of PLGA-NPs
ENP    = 0.055         ;unitless typical tumor fraction accessible by PTX free drug in the
 form of PLGA-NPs


S1=VPLMSC
S2=VT
S4=(1/FUPLNP)*VPLNP      ;FUPLNP: scaling factor PB ratio for PTX in the form of PLGA-NP
s
S5=VT
S7=(1/FUPL)*VPL          ;FUPL: scaling factor PB ratio for PTX free drug
S8=VT

A_0(10) = 5000/VPLMSC    ;initialize concentration in concentration summation in centra
l compartments
```

```
$DES
CPLMSC   = A(1)/VPLMSC
CTMSC    = A(2)/VT
CPHEMSC  = A(3)/VPHEMSC
CPLNP    = A(4)/VPLNP
CTNP     = A(5)/VT
CPHENP   = A(6)/VPHENP
CPL      = A(7)/VPL
CT       = A(8)/VT
CPHE     = A(9)/VPHE

DADT(1) = -K12*CPLMSC*VPLMSC-K13*CPLMSC*VPLMSC-KEXO*VPLMSC*CPLMSC-KREL*VPLMSC*CPLMSC

DADT(2) = K12*CPLMSC*VPLMSC-KEXO*VT*CTMSC-KREL*VT*CTMSC

DADT(3) = K13*CPLMSC*VPLMSC-KEXO*VPHEMSC*CPHEMSC-KREL*VPHEMSC*CPHEMSC

DADT(4) = CLDNP*(CPHENP-CPLNP)-CLNP*CPLNP-(2*PNP*RCAP)/(RKROGH*RKROGH)*(CPLNP*ENP-CTN
P)*VT-(6*DNP)/(RTUMOR*RTUMOR)*(CPLNP*ENP-CTNP)*VT-KREL*CPLNP*VPLNP+KEXO*VPLMSC*CPLMSC

DADT(5) = (2*PNP*RCAP)/(RKROGH*RKROGH)*(CPLNP*ENP-CTNP)*VT+(6*DNP)/(RTUMOR*RTUMOR)*(CP
LNP*ENP-CTNP)*VT-KREL*CTNP*VT+KEXO*VT*CTMSC

DADT(6) = CLDNP*(CPLNP-CPHENP)-KREL*CPHENP*VPHENP+KEXO*VPHEMSC*CPHEMSC

DADT(7) = CLD*(CPHE-CPL)-CL*CPL-(2*PDRUG*RCAP)/(RKROGH*RKROGH)*VT*(CPL*EDRUG-CT)-(6*DD
RUG)/(RTUMOR*RTUMOR)*VT*(CPL*EDRUG-CT)+KREL*CPLNP*VPLNP+KREL*VPLMSC*CPLMSC

DADT(8) = (2*PDRUG*RCAP)/(RKROGH*RKROGH)*(CPL*EDRUG-CT)*VT+(6*DDRUG)/(RTUMOR*RTUMOR)*
(CPL*EDRUG-CT)*VT+KREL*CTNP*VT+KREL*VT*CTMSC

DADT(9) = CLD*(CPL-CPHE)+KREL*CPHENP*VPHENP+KREL*VPHEMSC*CPHEMSC

DADT(10)= DADT(1)/S1+DADT(4)/S4+DADT(7)/S7  ;SUM OF CENT COMP CONC    NO MASS TRANSFER

DADT(11)= DADT(2)/S2+DADT(5)/S5+DADT(8)/S8  ;SUM OF TUMOR COMP CONC    NO MASS TRANSFER


CTOTC = A(1)/VPLMSC+A(4)/VPLNP+A(7)/VPL     ;total plasma concentration without account
 for BP ratio
CTOTT = A(2)/VT+A(5)/VT+A(8)/VT
```

```
$ERROR
IF(CMT.EQ.10) THEN
IPRED = A(1)/S1+A(4)/S4+A(7)/S7
Y = IPRED*(1+ERR(1))
ENDIF

IF(CMT.EQ.11) THEN
IPRED = A(2)/S2+A(5)/S5+A(8)/S8
Y = IPRED*(1+ERR(2))
ENDIF

$THETA
(0,1.17)          ;K12     1/hr
(0,8.24)          ;K13     1/hr
(0.081 FIX)       ;KEXO    1/hr
(0,0.0000000672)  ;VPLMSC  mL
(0,15800)         ;VPHEMSC mL

$OMEGA
0.769
0.339

$ESTIMATION METHOD=0 MAXEVAL=30000000 NOABORT NSIG=3 SIGL=9 PRINT=5 MSF=0011.msf
$COV
$TABLE ID TIME DV CMT MDV IPRED PRED CTOTC CTOTT CWRES ONEHEADER NOPRINT FILE=sdtab0011
$TABLE ID K12 K13 KEXO VPLMSC VPHEMSC ONEHEADER NOPRINT FILE=patab0011
```

PK-PD Model NONMEM code

Shen Cheng

11/6/2020

```
$PROB PKPD MODEL
$INPUT C ID TIME AMT DV CMT TV0 MDV EVID ORIDV TIMEDAY TYPE DVLOG CENSOR UPLL
$DATA CTR_PTX_NP_MSC_M3_3.csv IGNORE=C
$SUBROUTINES ADVAN13 TRANS=1 TOL=12

$MODEL NCOMP=10
COMP(CMSC)  ;central compartment for PTX in the form of nano-MSCs
COMP(TMSC)  ;tumor compartment for PTX in the form of nano-MSCs
COMP(PMSC)  ;peripheral compartment for PTX in the form of nano-MSCs
COMP(CNP)   ;central compartment for PTX in the form of PLGA-NPs
COMP(TNP)   ;tumor compartment for PTX in the form of PLGA-NPs
COMP(PNP)   ;peripheral compartment for PTX in the form of PLGA-NPs
COMP(CPTX)  ;central compartment for PTX free drug
COMP(TPTX)  ;tumor compartment for PTX free drug
COMP(PPTX)  ;peripheral compartment for PTX free drug
COMP(M1)    ;tumor compartment(fit with tumor bioluminescence)

$PK
K12    = 1.45         ;1/hr  rate constant describing central compartment to tumor comp
artment transfer for PTX in the form of nano-MSCs
K13    = 10.2         ;1/hr rate constant describing central compartment to peripheral
compartment transfer for PTX in the form of nano-MSCs
KEXO   = 0.081        ;1/hr first order exocytosis rate constant for PTX-PLGA-NPs from
nano-MSCs
VPLMSC = 0.000000071   ;mL central compartment volume of distribution for PTX in the fo
rm of nano-MSCs
VPHEMSC= 15021        ;mL peripheral compartment volume of distribution for PTX in the
form of nano-MSCs
RKROGH = 0.0008       ;cm average distance between two tumor associated capillaries
RCAP   = 0.0075       ;cm average radius of tumor associated capillaries
CL     = 0.909        ;mL/hr clearance for PTX free drug
CLD    = 0.336        ;mL/hr distribution Clearance for PTX free drug
VPL    = 6.64         ;mL volume of distribution of central compartment for PTX free dr
ug
VPHE   = 18.5         ;mL volume of distribution of peripheral compartment for PTX free
 drug
```

```
FUPL   = 0.0237        ;unitless plasma to blood ratio for PTX free drug
PDRUG  = 0.0875        ;cm/hr permeability rate constant for PTX free drug
DDRUG  = 0.01          ;cm^2/hr diffusion rate constant for PTX free drug
EDRUG  = 0.44          ;unitless tumor fraction accessible by PTX free drug
CLNP   = 0.241         ;mL/hr clearance for PTX in the form of PLGA-NPs
CLDNP  = 0.0627        ;mL/hr distribution clearance for PTX in the form of PLGA-NPs
VPLNP  = 1.32          ;mL volume of distribution of central compartment for PTX in the 
form of PLGA-NPs
VPHENP = 43.2          ;mL volume of distribution of peripheral compartment for PTX in t
he form of PLGA-NPs
FUPLNP = 0.003         ;unitless plasma to blood ratio for PTX in the form of PLGA-NPs
KREL   = 0.0085        ;1/hr PTX release rate constant
PNP    = 0.00035       ;cm/hr permeability rate constant for PTX in the form of PLGA-NPs
DNP    = 0.0000036     ;cm^2/hr diffusion rate constant for PTX free drug in the form of
 PLGA-NPs
ENP    = 0.055         ;unitless tumor fraction accessible by PTX free drug in the form 
of PLGA-NPs

MU_1   = LOG(THETA(1))         ;KMAXPTX
MU_2   = LOG(THETA(2))         ;KMAXNP
MU_3   = LOG(THETA(3))         ;KMAXMSC

;;;LKg0 START
IF(TYPE.EQ.1) LKg0   = LOG(THETA(4))       ;no treatment
IF(TYPE.EQ.2) LKg0   = LOG(THETA(5))       ;PTX Solution
IF(TYPE.EQ.3) LKg0   = LOG(THETA(6))       ;PTX PLGA NPs
IF(TYPE.EQ.4) LKg0   = LOG(THETA(7))       ;nano-MSCs
;;;LKg0 END
MU_4   = LKg0

;;;LTVBL START
IF (TYPE.EQ.1) LTVBL = LOG(THETA(8))       ;no treatment
IF (TYPE.EQ.2) LTVBL = LOG(THETA(9))       ;PTX Solution
IF (TYPE.EQ.3) LTVBL = LOG(THETA(10))      ;PTX PLGA NPs
IF (TYPE.EQ.4) LTVBL = LOG(THETA(11))      ;nano-MSCs
;;;LTVBL END
MU_5  = LTVBL

IC50PTX = 1.5 ;ng/mL concentration of PTX free drug can introduce 50% KMAXPTX
IC50NP  = 5.7 ;ng/mL concentration of PTX in the form of PLGA-NPs can introduce 50% KMA
XNP
```

```
KMAXPTX   = DEXP(MU_1 + ETA(1))   ;1/hr maximal tumor killing rate induced by PTX free d
rug
KMAXNP    = DEXP(MU_2 + ETA(2))   ;1/hr maximal tumor killing rate induced by PTX in the
 form of PLGA-NPs
KMAXMSC   = DEXP(MU_3 + ETA(3))   ;1/hr first order tumor killing rate constant induced
 by PTX in the form of nano-MSCs
Kg0       = DEXP(MU_4 + ETA(4))   ;1/hr tumor growth rate constant
TVBL      = DEXP(MU_5 + ETA(5))   ;1000000 photon/sec baseline tumor volume


A_0(10) = TVBL ;initialize tumor compartment

$DES
TV = A(10)

IF (TV.LE.0) TV=0.001 ;prevent the existance negative tumor bioluminescence

VT = 0.2749*(TV**0.2722)  ;mL convert tumor bioluminescence predicted to tumor volume

RTUMOR = (VT**(1/3))/1.6  ;cm calculate tumor radius based on tumor volume calculated

CPLMSC   = A(1)/VPLMSC
CTMSC    = A(2)/VT
CPHEMSC  = A(3)/VPHEMSC
CPLNP    = A(4)/VPLNP
CTNP     = A(5)/VT
CPHENP   = A(6)/VPHENP
CPL      = A(7)/VPL
CT       = A(8)/VT
CPHE     = A(9)/VPHE

DADT(1) = -K12*CPLMSC*VPLMSC-K13*CPLMSC*VPLMSC-KEXO*VPLMSC*CPLMSC-KREL*VPLMSC*CPLMSC

DADT(2) = K12*CPLMSC*VPLMSC-KEXO*VT*CTMSC-KREL*VT*CTMSC

DADT(3) = K13*CPLMSC*VPLMSC-KEXO*VPHEMSC*CPHEMSC-KREL*VPHEMSC*CPHEMSC

DADT(4) = CLDNP*(CPHENP-CPLNP)-CLNP*CPLNP-(2*PNP*RCAP)/(RKROGH*RKROGH)*(CPLNP*ENP-CTN
P)*VT-(6*DNP)/(RTUMOR*RTUMOR)*(CPLNP*ENP-CTNP)*VT-KREL*CPLNP*VPLNP+KEXO*VPLMSC*CPLMSC

DADT(5) = (2*PNP*RCAP)/(RKROGH*RKROGH)*(CPLNP*ENP-CTNP)*VT+(6*DNP)/(RTUMOR*RTUMOR)*(CP
```

```
LNP*ENP-CTNP)*VT-KREL*CTNP*VT+KEXO*VT*CTMSC

DADT(6) = CLDNP*(CPLNP-CPHENP)-KREL*CPHENP*VPHENP+KEXO*VPHEMSC*CPHEMSC

DADT(7) = CLD*(CPHE-CPL) -CL*CPL-(2*PDRUG*RCAP)/(RKROGH*RKROGH)*VT*(CPL*EDRUG-CT)-(6*D
DRUG)/(RTUMOR*RTUMOR)*VT*(CPL*EDRUG-CT)+KREL*CPLNP*VPLNP+KREL*VPLMSC*CPLMSC

DADT(8) = (2*PDRUG*RCAP)/(RKROGH*RKROGH)*(CPL*EDRUG-CT)*VT+(6*DDRUG)/(RTUMOR*RTUMOR)*
(CPL*EDRUG-CT)*VT+KREL*CTNP*VT+KREL*VT*CTMSC

DADT(9) = CLD*(CPL-CPHE)+KREL*CPHENP*VPHENP+KREL*VPHEMSC*CPHEMSC

CTOTC = A(1)/VPLMSC+A(4)/VPLNP+A(7)/VPL   ;total plasma drug concentration without acco
unting for BP ratop in plasma
CTOTT = A(2)/VT    +A(5)/VT   +A(8)/VT

KkillPTX = KMAXPTX*CT/(IC50PTX + CT)

KkillNP  = KMAXNP*CTNP/(IC50NP + CTNP)

KkillMSC = KMAXMSC*CTMSC

DADT(10) =  Kg0*A(10)-KkillPTX*A(10)-KkillNP*A(10)-KkillMSC*A(10)

$ERROR
AA1  = A(1)
AA2  = A(2)
AA3  = A(3)
AA4  = A(4)
AA5  = A(5)
AA6  = A(6)
AA7  = A(7)
AA8  = A(8)
AA9  = A(9)
AA10 = A(10)

Ew = A(10)

IF (Ew.LE.0) Ew=0.001 ;prevent the existance negative tumor bioluminescence

;;;DUMMY VARIABLE START
    TYPE1=0
```

```
    TYPE2=0
    TYPE3=0
    TYPE4=0

    IF(TYPE.EQ.1) TYPE1=1   ;no treatments
    IF(TYPE.EQ.2) TYPE2=1   ;PTX Solution
    IF(TYPE.EQ.3) TYPE3=1   ;PTX PLGA NPs
    IF(TYPE.EQ.4) TYPE4=1   ;nano-MSCs
;;;DUMMY VARIABLE END

;;;SDSL START
    SDSL1=THETA(12)      ;proportional error for no treatment group
    SDSL2=THETA(13)      ;proportional error for PTX solution group
    SDSL3=THETA(14)      ;proportional error for PTX PLGA NPs group
    SDSL4=THETA(15)      ;proportional error for nano-MSCs group

    SDSL=SDSL1*TYPE1+SDSL2*TYPE2+SDSL3*TYPE3+SDSL4*TYPE4
;;;SDSL END

UPL=UPLL  ;set upper limit for each animal
IPRED = Ew
IF (COMACT==1) PREDV = IPRED       ;carryout PRED

   AQL=1
   IF (DV>UPL) AQL=2
   IF (MDV==1) AQL=0

IF (AQL.EQ.1) THEN
 F_FLAG=0
 Y=IPRED + IPRED*SDSL*ERR(1)       ;ELS for observations below quantification limit
ENDIF

IF (AQL.EQ.2)THEN
 F_FLAG=1
 Y=1-PHI((UPL-IPRED)/(SDSL*IPRED)) ;MLE for observations above quantification limit
 MDVRES=1
ENDIF


$THETA
(0,0.0031)       ;KMAXPTX          1
(0,0.0014)       ;KMAXNP           2
```

```
(0,0.000001)     ;KMAXMSC          3

(0,0.0034)       ;Kg0 no treatment  4
(0,0.0036)       ;Kg0 PTX solution  5
(0,0.0042)       ;Kg0 PTX PLGA NPs  6
(0,0.0049)       ;Kg0 nano-MSCs     7

(0,0.36)         ;TVBL no treatment 8
(0,0.25)         ;TVBL PTX solution 9
(0,0.6)          ;TVBL PTX PLGA NPs 10
(0,0.1)          ;TVBL nano-MSCs    11

(0.5)            ;ERR no treatment  12
(0.7)            ;ERR PTX solution  13
(0.6)            ;ERR PTX PLGA NPs  14
(0.7)            ;ERR nano-MSCs     15

$OMEGA
0 FIX  ;KMAXPTX    1
0 FIX  ;KMAXNP     2
0 FIX  ;KMAXMSC    3
0 FIX  ;Kg0        4
0.7    ;TVBL       5


$SIGMA
1 FIX

$EST METHOD=1 INTER LAPLACIAN NUMERICAL SLOW MAXEVAL=9999 NOABORT NSIG=3 SIGL=9 PRINT=5
 MSF=0050.msf
$COV SLOW

$TABLE ID TIME DV TYPE TVBL Kg0 KMAXPTX KMAXNP KMAXMSC
KkillMSC KkillPTX KkillNP CT CTNP CTMSC CTOTT TV VT RTUMOR IC50PTX IC50NP
AA1 AA2 AA3 AA4 AA5 AA6 AA7 AA8 AA9 AA10
IPRED PRED PREDV CWRES ONEHEADER NOPRINT FILE=sdtab0077
$TABLE ID TYPE TVBL Kg0 KMAXPTX KMAXNP KMAXMSC
ONEHEADER NOPRINT FILE=patab0077
```
